# Movement and habitat selection of a large carnivore in response to human infrastructure differs by life stage

**DOI:** 10.1186/s40462-022-00349-y

**Published:** 2022-11-29

**Authors:** N. H. Thorsen, J. E. Hansen, O.-G. Støen, J. Kindberg, A. Zedrosser, S. C. Frank

**Affiliations:** 1 Norwegian Institute for Nature Research, Oslo, Norway; 2 Faculty of Environmental Sciences and Natural Resource Management, Norwegian University of Life Sciences, Ås, Norway; 3 Faculty of Technology, Natural Sciences and Maritime Sciences, University of South-Eastern Norway, Bø, Telemark Norway; 4 Department of Wildlife, Fish and Environmental Studies, Swedish University of Agricultural Sciences, Umeå, Sweden; 5 Department of Integrative Biology, Institute of Wildlife Biology and Game Management, University of Natural Resources and Applied Life Sciences, Vienna, Austria

**Keywords:** Human disturbance, Human-modified, Roads, Settlements, Life stage, Home range, Dispersal, Brown bear, Connectivity

## Abstract

**Background:**

The movement extent of mammals is influenced by human-modified areas, which can affect population demographics. Understanding how human infrastructure influences movement at different life stages is important for wildlife management. This is true especially for large carnivores, due to their substantial space requirements and potential for conflict with humans.

**Methods:**

We investigated human impact on movement and habitat selection by GPS-collared male brown bears (*Ursus arctos*) in two life stages (residents and dispersers) in central Sweden. We identified dispersers visually based on their GPS locations and used hidden Markov models to delineate dispersal events. We used integrated step selection analysis (iSSA) to infer movement and habitat selection at a local scale (availability defined by hourly relocations), and resource selection functions (RSFs) to infer habitat selection at a landscape scale (availability defined by the study area extent).

**Results:**

Movement of residents on a local scale was facilitated by small forestry roads as they moved faster and selected areas closer to forestry roads, and they avoided areas closer to larger public roads and buildings on both scales. Dispersers were more ambivalent in their response to human infrastructure. Dispersers increased their speed closer to small forestry roads and larger public roads, did not exhibit selection for or against any road class, and avoided areas closer to buildings only at local scale. Dispersers did not select for any features on the landscape, which is likely explained by the novelty of the landscape or their naivety towards it.

**Conclusion:**

Our results show that movement in male brown bears is life stage-dependent and indicate that connectivity maps derived from movement data of dispersing animals may provide more numerous and more realistic pathways than those derived from resident animal data alone. This suggests that data from dispersing animals provide more realistic models for reconnecting populations and maintaining connectivity than if data were derived from resident animals alone.

**Supplementary Information:**

The online version contains supplementary material available at 10.1186/s40462-022-00349-y.

## Introduction

Human activity and infrastructure have reduced the movement extent of wildlife globally [1]. Animals are generally sensitive to human infrastructure [2], especially to the creation of linear structures [3], which is commonly reflected in their movement patterns. Maintaining connectivity, i.e. the ease of movement between suitable habitat patches or between populations, within a human-dominated landscape is important to avoid fragmentation of populations and to ensure gene flow [4, 5]. As dispersal contributes to population connectivity and genetic diversity [6], it is crucial to understand how dispersing individuals (“dispersers”) respond to human infrastructure and if they respond differently compared with individuals settled within a home range (“residents”). Connectivity is often derived from habitat selection estimates [4]. Habitat selection can vary across life stages [7, 8], i.e. dispersers compared with residents. Thus, connectivity estimates may differ depending on which life stage the habitat selection estimates are obtained from [8].

Understanding how movement decisions differ by life stage is important for the conservation of species, e.g., for defining potential connectivity and conservation corridors within and between populations [9]. Dispersal can be risky and energetically costly, as it often exposes individuals to unknown environments, especially in human-modified landscapes [10–12] where mortality risk can be higher [13]. This implies that dispersers are either unable to perceive human risk or fail to adjust habitat use or movement in response to human risk due to their naivety [14] or they might be more ‘tolerant’ or ‘bold’ and traverse risky habitats [15]. In contrast, the home range is familiar to a resident, and risk encountered during movement may be mitigated through spatiotemporal shifts or altered habitat selection based on prior experience [16, 17]. This strategy might not be available to naïve dispersers facing unexpected or less predictable, risky features on the human-dominated landscape [18].

Habitat selection, i.e. the disproportionate use of a habitat feature in relation to its availability [19], can be estimated at different spatial scales depending on how availability is defined. Throughout this article we refer to habitat selection on the “landscape scale” when the availability is defined for an area many times the size of an animal’s home range, e.g. an entire study area (cf. second order habitat selection [19]). We refer to habitat selection at the “local scale” when the availability is defined over smaller areas or shorter distances (e.g. for step selection functions) that an animal is able to traverse between successive (e.g. hourly) locations (cf. forth order habitat selection [19]). Animals may respond differently to the same covariate depending on the scale of availability [20, 21]. This also applies to human infrastructure, e.g. wolves (*Canis lupus*) avoid gravel roads within their home range but select gravel roads on a local scale [22]. Spatial scale can also be of importance for movement and habitat selection during different life stages, because dispersers navigating a novel landscape will likely only know what is in its immediate surroundings and have less knowledge at a landscape scale.

Here we use the brown bear (*Ursus arctos*) as a model species within a human-modified landscape in Sweden to study the impact of human infrastructure, i.e. roads and buildings, movement and habitat selection at the landscape and local scale during two life stages. Like other large carnivores, brown bears have large home ranges [23] and can travel long distances [24]. There is ample scientific evidence that humans influence brown bear behaviour [25–27], and bears are able to perceive and respond to local context-specific risks [28], but it is unknown how dispersers navigate human-modified environments compared with residents. In this study, we focus on male brown bears in two life stages, dispersers, and residents. We estimate habitat selection and movement at the landscape and local scale and evaluate whether the effect of human infrastructure differs between the two life stages.

We hypothesize (H1) that human infrastructure influence movement of dispersing and resident male brown bears. In support of (H1), we predict (P1) that the most parsimonious model explaining movement and habitat selection for both dispersers and residents will include buildings or roads at one or both spatial scales. We hypothesize (H2) that dispersers will be more naïve or risk-tolerant compared with residents. In support of H2, we predict (P2) that dispersers will show less avoidance of or be closer to buildings and roads compared with residents. We hypothesize (H3) that bears of both life stages will be more sensitive to human infrastructure at the local scale than the landscape scale. In support of H3, we predict (P3) that bears in each life stage will exhibit stronger avoidance of buildings and roads at the local scale compared to the landscape scale.

## Methods

### Study area and study species

**Fig. 1:**
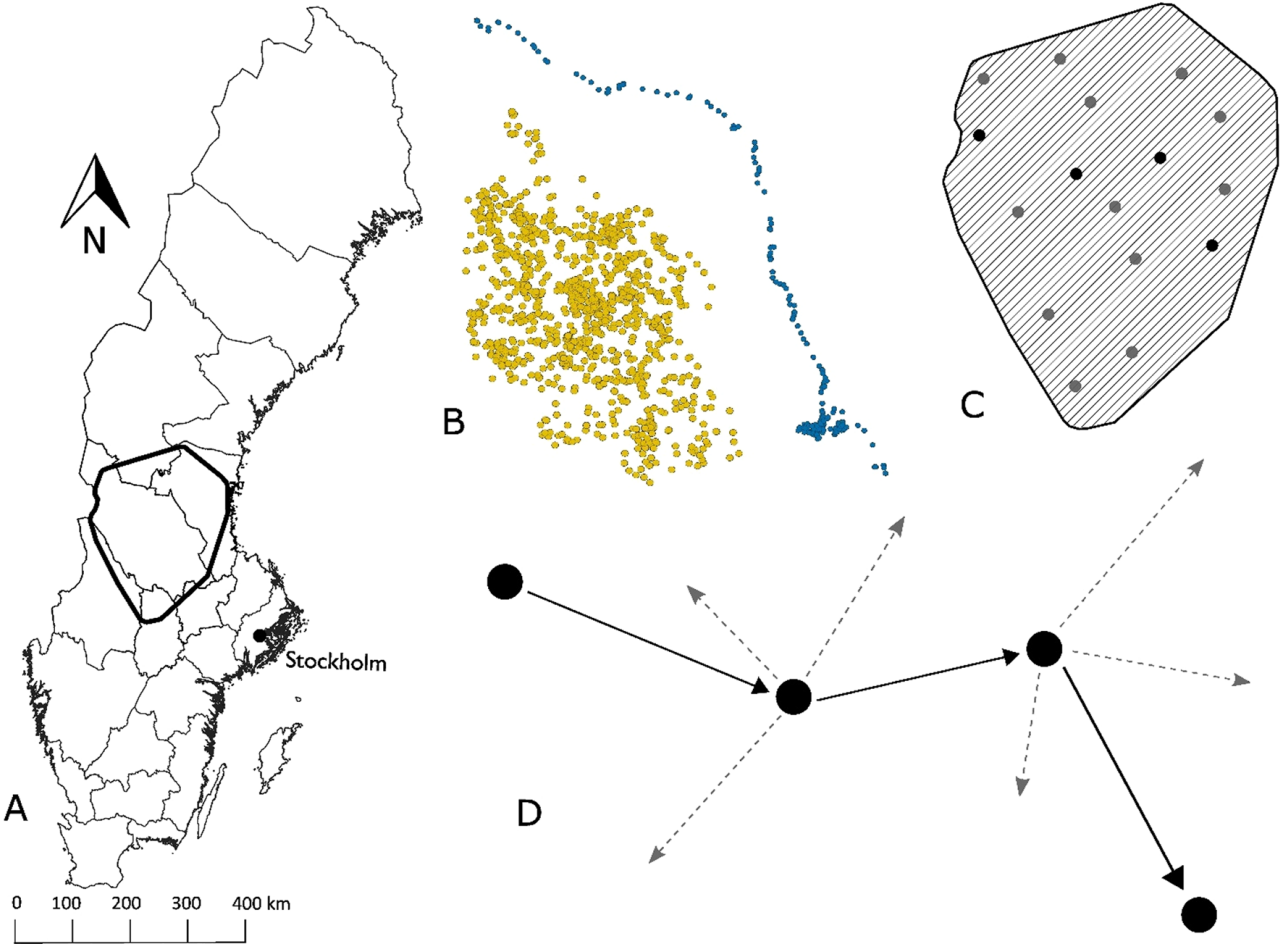
Diagram shows **A** study area within Sweden. **B** Differences between GPS locations of resident (gold) and dispersing (blue) male brown bears. **C** Availability space and sampling design for landscape scale for resource selection function (RSF) where grey dots represent available location and black points represent used. **D** Availability and sampling design for local scale for integrated step selection analysis (iSSA), where grey arrows represent available steps and black arrows represent used steps

The study area is located in southcentral Sweden (approximately 61° N, 15° E), primarily within Gävleborg and Dalarna counties, spanning ~ 50,000 km^2^ (Fig. 1A). The landscape consists of boreal forest, bogs, lakes, and sparse agricultural land. The intensively managed forest is dominated by Norway spruce (*Picea abies*) and Scots pine (*Pinus sylvestris*) [29]. Rolling hills comprise the general topography, with steeper and more rugged terrain in the western portion of the study area (elevation range 0–997 m a.s.l.). Human settlement in the area is sparse, with an average of 8.64 inhabitants/km^2^ and people tend to live in small villages [30]. There are several larger towns and cities, but urban areas and sub-urban settings contribute to small fractions of our study area and few bears are exposed to this magnitude of human infrastructure. There is an extensive road network, dominated by gravel roads used for forestry. The road density is low in a European context [31] and the traffic volumes are low in a Swedish perspective. Almost all roads are unfenced, only the largest public roads are fenced.

Current brown bear density in the study area ranges from ca 20 to 60 bears per 1000 km^2^ [32]. Scandinavian brown bears are subject to high hunting pressure and approximately 70% of the total mortality is due to legal hunting [32]. They generally avoid humans and their settlements [33]. By way of human infrastructure, brown bears are exposed to mostly roads and buildings in our study area. Male brown bears have large home ranges, on average 800 km^2^ [34], encompassing all types of human infrastructure, which they generally avoid [35]. We focused on males because dispersal is primarily male-biased (94%) [36].

### Telemetry data

Brown bears were captured, collared, and monitored from 2007 to 2017 as part of a long-term research project [37]. See Arnemo and Evans [38] for a more detailed description of capture and handling procedures. Capture and handling of bears was conducted by permit under Swedish authorities and ethical committees. Bears were fitted with GPS collars (GPS Plus, Vectronic Aerospace GmbH) with different programming schedules, but all were scheduled to acquire at least one GPS location each hour. All GPS locations were resampled to one location every hour (± 3 min tolerance). We retained only GPS locations with a dilution of precision (DOP) of less than 10 to reduce location error [39]. All GPS locations overlaying water bodies were removed prior to analyses. As our focus was on movement and habitat selection in relation to human infrastructure, we removed locations associated with resting sites (day and night beds) [28]. We defined a bed site as a cluster of GPS locations with a maximum distance of 50 m between any two GPS locations in the cluster, a maximum distance of 30 m between two consecutive GPS locations and at least 5 consecutive locations, i.e., the bear had to spend at least 4 h in the same location to be defined as a bed site.

### Defining dispersing and resident bear-years

We focused only on natal dispersal, i.e. the permanent movement from birth site to first breeding, and will hereafter refer to it as dispersal. We visually examined the GPS tracks of every bear-year, i.e. the unique combination of bear ID and year, to identify bear-years with diagnostic linear tracks typical of dispersal events (Fig. 1B). This approach might underestimate dispersal in males that gradually move away from their maternal range over multi-year periods, however, this behavior is difficult to disentangle from home range drift or infidelity [40]. In addition, previous studies have detected high rates (> 92%) of male dispersal [36, 41]. We performed ‘path segmentation’ [42] on movement tracks of bear-years identified with a dispersal event and used hidden Markov models (HMM), a form of state-space modeling [43], to define the transient period of dispersal. Hence, this method identified the onset and end of the dispersal event. For each track, we fit seven HMMs that varied in the number of states and the initial parameters (see Additional file 1: supplement S1 for more details on model fitting and structures). We selected the most parsimonious (hereafter ‘best’) model using Akaike’s Information Criterion [44] and used the Viterbi algorithm [45] to classify behaviors from the best model. Based on the classified behaviors, we defined the onset and end for the dispersal period for the bear-years identified as dispersing (Additional file 1: supplement S1). Only one dispersal period was defined for each of the bear-years identified as dispersing, and only data from this period for each bear-year was used in the further analysis for dispersers. We used the R package ‘moveHMM’ [46] for fitting HMMs, model selection, and behavioral classification. Dispersal phases lasted from 21 to 65 days (mean: 43 ± 15) for the 15 males (15 bear-years) defined as dispersing. During their dispersal events, these bears ranged in age from 2 to 4 years old (mean 2.7 years, n = 15).

We defined resident bear-years as all years that a GPS-collared bear had been solitary for at least three years (Fig. 1B), and no dispersal event had been detected. Males separate from their mother at 1 or 2 years of age [47] and Støen, Zedrosser [41] showed that 92.3% of male bears (n = 67) had dispersed by four years of age in the study area, with no observed dispersal events at 4 years of age. Hence, based on our definition of a resident bear-year, we avoided the inclusion of solitary pre-dispersing males, i.e. young males that had left their mother but not dispersed yet, as resident bear-years. We defined an active period between 25 April and 20 August which follows den emergence and precedes the hunting season for brown bears in Sweden. Brown bears in Sweden are known to change their movement pattern after the onset of hunting [48]. For residents, we excluded all data outside this period, and only individuals with GPS locations covering at least 70% of the days during the active period each year for further analysis. For dispersers there were two cases which extended outside this period: one started dispersing 21 April and another whose dispersal ended 22 August. To maximize disperser sample size, we included the six days of data outside the active period for these two individuals. We identified 20 males spanning 46 bear-years that met our resident criteria. The resident bear-years ranged in age from 4 to 21 years old (mean = 9.7 years, n = 45, one with unknown age but classified as adult based on head circumference).

### Covariates

We included the following ‘core’ covariates reported to influence brown bear habitat selection and movement in our analysis: terrain ruggedness index (TRI) [35], clearcuts [49], bogs [50], and distance to water [51, 52]. We calculated TRI from a digital elevation model (25-m resolution) with the R package spatialEco [53] using a 3 × 3 cell moving window. We obtained data on landcover, roads, and buildings from the Swedish Mapping, Cadastral and Land Registration Authority [54]. In Sweden, forestry practitioners must report timber harvesting activities [55], and we used the data for defining clearcuts as logged areas from first cutting up to 10 years [56]. We created a clearcut raster (presence = 1, absence = 0) for each year of the study.

We divided our road data into forestry and public roads. Forestry roads represent the majority of the road network in the study area (mean = 1.27 ± 1.07 km/km^2^), which are mainly small gravel roads built for forestry and usually open for the public. Public roads are larger and mostly paved and associated with higher traffic volume (mean = 0.18 km/km^2^, SD = 0.45). We included all buildings, the majority of which were houses or cabins (mean density = 10.7 ± 39.4 buildings/km^2^). We rasterized all covariates to a resolution of 25 m. We calculated the Euclidean distance from all cells in the raster to the nearest forestry road, public road, and building, for each feature separately. All distance to covariates were log transformed prior to analysis to attenuate covariate effects at longer distances.

### Habitat selection and movement analyses

We used exponential resource selection functions (RSF) to estimate habitat selection at the landscape scale (availability defined by the study area extent) [57], and integrated step selection analysis (iSSA) to estimate habitat selection and movement parameters at the local scale (availability defined by hourly relocations) [58]. We used the bear locations excluding bed sites for RSF and iSSA. RSF and iSSA models were fitted at the bear-year level, i.e. one model for each bear-year. The estimates from each model on the bear-year level were later averaged to obtain one population estimate for dispersing individuals and one for resident individuals.

For the RSF, the availability space was defined as the 100% minimum convex polygon of all observed GPS locations for all bear-years buffered by the radius of a circular mean male home range size (r =  ~ 18 km). For each bear-year, we randomly sampled available GPS locations from the availability space with a ratio of 20:1 available-to-use (Fig. 1C). The RSF was obtained by fitting a generalized linear model with the glm function in R. The GPS locations were used as the response variable and coded ‘1’ for used and ‘0’ for available.

An iSSA is a form of step selection functions that simultaneously estimate habitat selection and movement parameters [58]. We created “steps” by combining two consecutive GPS locations that were not part of a bed site. An iSSA requires at least two valid consecutive steps, as turning angles need to be calculated and included in model structure. Every step had a duration of 1 h. An iSSA uses ‘local scale availability’, i.e. locations to which an animal could possibly have moved to in a given step. Based on the used steps, we calculated step lengths and fitted a gamma probability density function based on maximum likelihood estimation for both life stages combined. We drew 20 step lengths from the fitted gamma distribution for each used step (20:1 available-to-use ratio) and combined them with turning angles drawn from a uniform distribution to generate ‘available’ steps (Fig. 1D). We assigned a unique stepID to each used step and its 20 associated available steps. The available steps represent what was locally available to bears at the starting point of every step. The iSSA was modelled using conditional logistic regression with used (coded as 1) and available steps (coded as 0) as the response variable, and the stepID as the stratum (for matching the used and available steps). Covariates for used and available steps were extracted at the start- and endpoints of the step. The covariates extracted at endpoints are used to infer habitat selection, while the covariates extracted at starting points together with an interaction with step length (or the logarithm of step length) are used to infer movement speed. See Avgar, Potts [58] for a detailed description of iSSA.

### Candidate models and model selection

For each RSF and iSSA, we developed candidate models for all dispersers and residents. Each candidate model set contained a model including a set of ‘core covariates’. Core covariates account for habitat features which previously have been shown to be important for bear habitat selection and movement. We chose the combination of core covariates as they should cover the most prevalent habitat classes in our study area. The core model was extended with additional covariates representing specific human infrastructure attributes to form competing candidate models. We also included a ‘full’ model containing all covariates into the analysis (Table 1). RSF and iSSA candidate model formulae were identical, except iSSA models included movement-related covariates (Table 1). For iSSA, we included step lengths (SL) and the natural logarithm of step lengths (lnSL) in all models to capture movement differences between life stages. All covariates were standardized with the formula *(X* – *mean of X*)/*standard deviation of X* and checked for collinearity. The highest correlation was 0.30, reported for the variables distance to public roads and distance to buildings.

**Table 1:** Candidate models used to test the relative importance of forestry roads, public roads, and buildings and habitat selection and movement in male brown bears

Model	Explanatory covariates
*Resource selection function (landscape availability)*	
Core	Clearcut + bog + TRI + Dist.Water
Forestry roads	Core + Dist.ForestryRoads
Public roads	Core + Dist.PublicRoads
Buildings	Core + Dist.Building
Forestry and public roads	Core + Dist.ForestryRoads + Dist.PublicRoads
Forestry roads and buildings	Core + Dist.ForestryRoads + Dist.Building
Public roads and buildings	Core + Dist.PublicRoads + Dist.Building
Full	Core + Dist.ForestryRoads + Dist.PublicRoads + Dist.Building
*Integrated step selection analysis (local availability)*	
Core	SL^a^ + log(SL) + cos(TA^b^) + clearcut_end^c^ + bog_end + TRI_end + Dist.Water_end^d^ + log(SL):clearcut_start
Forestry roads	Core + Dist.ForestryRoads_end + log(SL): Dist.ForestryRoads_start
Public roads	Core + Dist.PublicRoads_end + log(SL): Dist.PublicRoads_start
Buildings	Core + Dist.Building_end + log(SL): Dist.Buildling_start
Forestry and public roads	Core + Dist.ForestryRoads_end + log(SL):Dist.ForestryRoads_start + Dist.PublicRoads_end + log(SL): Dist.PublicRoads_start
Forestry roads and buildings	Core + Dist.ForestryRoads_end + log(SL): Dist.ForestryRoads_start + Dist.Building_end + log(SL): Dist.Buildling_start
Public roads and buildings	Core + Public roads + log(SL): Dist.PublicRoads_start + Dist.Building_end + log(SL): Dist.Buildling_start
Full	Core + Dist.ForestryRoads_end + log(SL): Dist.ForestryRoads_start + Dist.PublicRoads_end + log(SL): Dist.PublicRoads_start + Dist.Building_end + log(SL): Dist Buildling_start

^a^Step length

^b^Turning angle

^c^“_end” and “_start” denote if the covariate was extracted from the start or the end point

^d^“Dist” is an abbreviation for “distance-to”, and the distance to features were log transformed

We performed model selection for each bear-year for both the RSF and iSSA models. We calculated AIC for all models for each bear-year and calculated the delta AIC from the best model for all candidate models within bear-years. We summed the delta AIC for all the candidate models and considered the model with the lowest mean AIC for each life stage as the best model for that life stage. Note that this may cause the best model to have a mean delta AIC > 0, because that model structure might not be ‘best’ across all bear-years for a given life stage and scale.

### Population level effects

To infer habitat selection and movement responses at the population level, i.e. for dispersers and residents, we averaged the bear-year models using inverse variance-weighted linear modelling [59], following the approach by Dickie, McNay [60]. We fitted inverse-variance linear regression models separately for residents and dispersers and for each RSF and iSSA model set. We used either RSF or iSSA coefficients as response variables and included the mean availability of each variable as an explanatory variable for a given bear-year to control for a possible functional response [61]. We used the inverse of the estimated variance for the coefficients as weights. The availability used in the inverse variance-weighted linear regression models was centered (x—mean of x), to aid in interpretation. The population-level coefficients can be interpreted as the mean coefficient at the mean availability for the males in the population (each for dispersers and residents and for the RSF and iSSA). We interpreted coefficients with 95% CIs overlapping with zero as ‘indifferent’ and non-overlapping 95% CIs as significant avoidance or selection, or as an effect of the covariate on movement rate [60]. We also recorded the direction of the coefficients for all individual bear-year models and reported the proportion of bear-year models that followed the same direction as the mean of all bear-years in a given life stage. This measure reflects the consistency of individual responses to covariates for each life stage between the local and the landscape scale for the population.

In iSSA, the estimated coefficients for SL, lnSL, and their interactions function as modifiers for the initial estimates of the scale and shape parameters, respectively, in the fitted gamma distribution (used for sampling the available step lengths) on step lengths [58]. For each bear-year, we adjusted the shape and scale parameters of the fitted gamma distribution. We calculated movement rates at the bear-year level by multiplying the adjusted shape and adjusted scale parameters from the gamma distribution. To illustrate changes in movement rates, we calculated movement rates at several levels for each of the focal covariates and kept all other interacting covariates (with lnSL) constant at their mean observed step value. Movement rates at the population level were obtained by calculating the mean of individual bear-year movement rates across the different levels of a given focal variable and for each life stages separately (iSSA only).

For both RSF and iSSA results, we calculated the relative selection strength (RSS) following Avgar, Lele [62] for all covariates in each analysis. The RSS was calculated based on the population-averaged estimates from the inverse-variance weighted linear models (one for each life stage and availability scale). For step selection functions (iSSA), the ln RSS is a relative measure of how likely the individual is to select a step that ends at location x_1_ in relation to a step that ends at location x_2_ (the reference location). For “distance to feature” covariates, we calculated the RSS moving one mean step length closer to the feature compared with staying at the same location, and for the other covariates we calculated the RSS of selecting a given feature over the mean of the covariate.

All distance calculations were performed in GRASS 7.2 [63]. We used the ‘amt’ package [64] for iSSA and R 3.6.0 all other statistical analyses [65].

## Results

The bed removal procedure removed 28% of the GPS locations, leaving 70,008 GPS locations for statistical analysis (8012 GPS locations for dispersers and 61,996 GPS locations for residents). The bed removal procedure biased removal of more observations during day (Additional file 2: Fig. S1). Mean step lengths were 729 m for dispersers and 586 m for residents. Used locations rarely (< 7%) occurred in bogs and clearcuts (Table 2), and the mean distance to water was 705 m for dispersers and 712 m for residents. There were large differences between dispersers and residents in the mean values of their used locations for human covariates (Table 2). Used locations of dispersers and residents occurred on average 788 and 1186 m from buildings, 3014 and 5213 m from public roads, and 250 and 294 m from forestry roads, respectively.

**Table 2:** Values for covariates at used locations for brown bears defined as residents and dispersers in Sweden

Covariate	Residents	Dispersers
Bogs	0.06 (0.03)	0.04 (0.03)
Clearcuts	0.06 (0.01)	0.06 (0.05)
Distance to water	712 (209)	705 (168)
Terrain ruggedness (TRI)	6.03 (1.38)	5.44 (1.47)
Distance to buildings	1186 (232)	788 (261)
Distance to public roads	5213 (2325)	3014 (1458)
Distance to forestry roads	294 (90)	250 (64)

The values in the table represent population means as calculated from the mean values for each bear year, with the standard error in the parentheses

The full models had the lowest mean delta AIC at both spatial scales for dispersers (landscape scale ΔAIC = 0.82, local scale ΔAIC = 2.06) as well as residents (landscape scale ΔAIC = 0.32, local scale ΔAIC = 0.96). The full model scored the lowest AIC for 40% and 20% of the bear-years for the dispersers at the landscape scale and local scale, respectively (Table 3). The full model scored the lowest AIC for 78% and 63% of the bear-years for the residents at the landscape scale and local scale, respectively (Table 3). The four best RSF (landscape scale) models for both life stages contained the covariate public roads, whereas the four best iSSA models (local scale) for both life stages contained the covariate forestry roads.

**Table 3:** Model selection for dispersing and resident male brown bears of model sets fit using resource selection functions (RSFs) and integrated step selection analysis (iSSA)

Model	Resident		Dispersal	
	Mean ∆AIC	Minimum AIC tally	Mean ∆AIC	Minimum AIC tally
*Landscape scale (RSF)*				
Full	**0.32**	**36 (0.78)**	**0.82**	**6 (0.40)**
Public roads and buildings	21.11	6 (0.13)	6.24	6 (0.40)
Forestry roads and public roads	189.84	0 (0)	20.88	1 (0.07)
Public roads	204.26	0 (0)	26.67	0 (0)
Forestry roads and buildings	536.49	4 (0.09)	33.84	0 (0)
Buildings	556.54	0 (0)	39.46	1 (0.07)
Forestry roads	1019.13	0 (0)	74.65	1 (0.07)
Core	1035.81	0 (0)	81.20	0 (0)
*Local scale (iSSA)*				
Full	**0.96**	**29 (0.63)**	**2.06**	**3 (0.2)**
Forestry roads and public roads	11.38	7 (0.15)	3.62	3 (0.2)
Forestry roads and buildings	11.47	5 (0.11)	6.74	4 (0.27)
Forestry roads	22.82	2 (0.04)	9.88	2 (0.13)
Public roads and buildings	40.22	2 (0.04)	15.07	0 (0)
Public roads	49.80	0 (0)	17.42	2 (0.13)
Buildings	50.68	1 (0.02)	19.95	0 (0)
Core	61.24	0 (0)	24.57	1 (0.07)

Bold denote the best models for different life stages and scale

Mean ∆AIC is the mean ∆AIC for all bear-years during the given life stage (n = 15 for dispersing males and n = 46 for resident males). Minimum AIC tally is the number of times that a given model had the lowest AIC among candidate models within a bear-year and the proportion of the model-specific tally for model sets is given in parentheses

### Core covariates

**Fig. 2:**
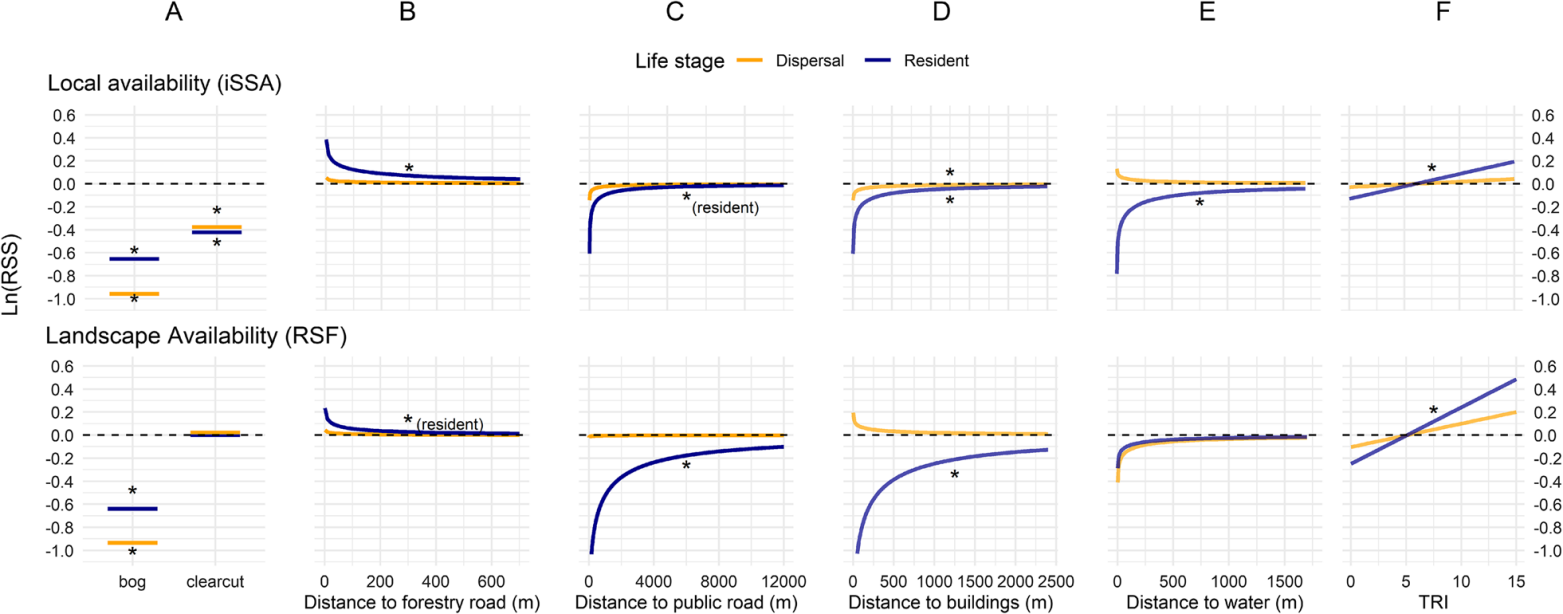
Relative selection strength (RSS) for dispersing and resident male brown bears from the inverse-variance weighted linear model. Column **A** is the RSS of staying in the habitat reference category (i.e. not bogs or clearcuts) compared with selecting for either bogs or clearcuts. Columns **B**, **C**, **D** and **E** show the RSS of moving closer towards the features (leftward) versus staying at the same distance across a range of starting distances (x-axis). Column **F** illustrates the RSS of selecting a given TRI value (x-axis) over the mean value (4.19). * indicates covariate was significant for respective ‘Resident’ and ‘Dispersal’ models (95% CIs did not overlap zero) for the curve that it is nearest. In cases where placement is not obvious, clarification is given in parenthesis

**Fig. 3:**
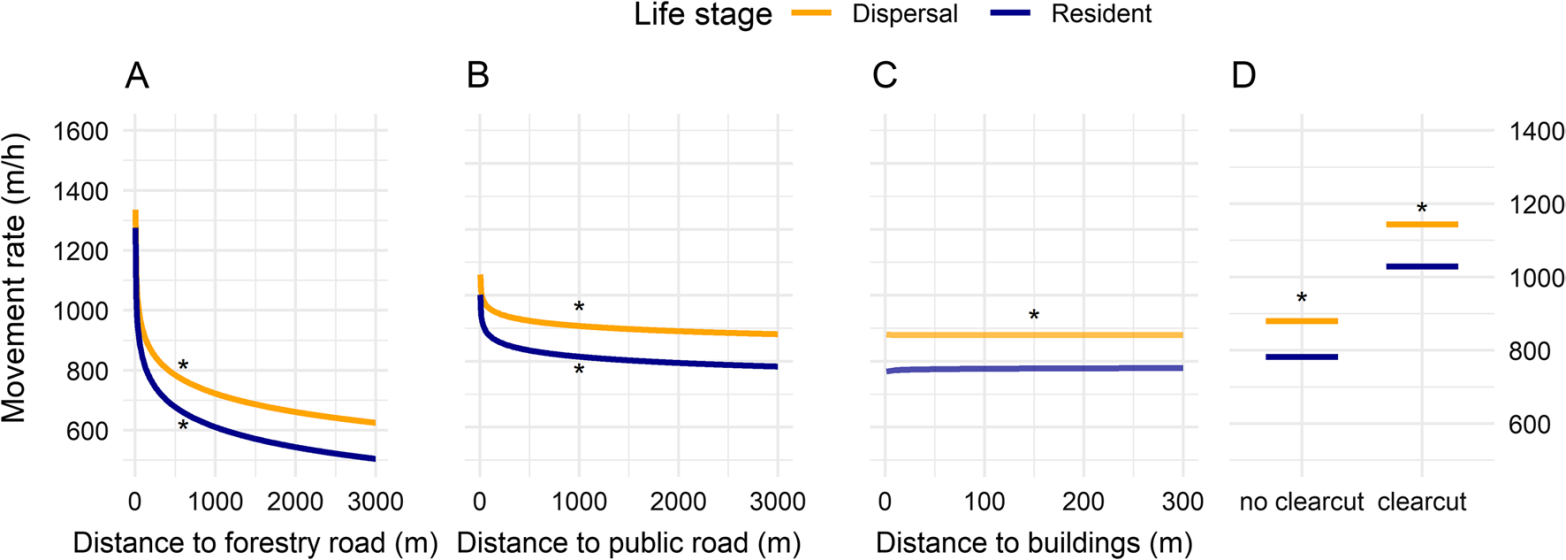
Mean movement rates (in meters/hour) for dispersing and resident male brown bears in relation to **A** distance to forestry roads, **B** distance to public roads with the same range as for forestry roads, **C** distance to buildings, **D** and whether or not the bear started in a clearcut. The focal variable has been varied while all other variables have been kept constant the mean of all used starting points and outside a clearcut for **A**, **B** and **C**. The asterisks * near ‘Resident’ and ‘Dispersal’ curves indicate that respective model estimates were significant (95% CIs did not overlap zero)

At the population level, dispersers and residents avoided bogs at both spatial scales, showing the same pattern for > 91% of the bear-years in each model (Table 4, Additional file 2: Fig. S2). Both dispersers and residents avoided clearcuts locally but were indifferent to them on the landscape scale (Table 4; Fig. 2). Dispersers and residents were indifferent to distance to water on the landscape scale, but residents selected for distances farther from water at the local scale (Table 4; Fig. 2). Dispersers were indifferent to TRI at either scale, however, residents selected for higher TRI at both scales (Table 4). In general, dispersers showed more individual variation and less consistency in their habitat selection towards the core covariates, i.e. a lower proportion of all bear-year estimates conformed to the same direction as the population mean effect, for dispersers compared with residents (Table 4; dispersers: mean of all proportions = 0.59, range = 0.333–0.933; residents: mean of all proportions = 0.79, range = 0.391–0.957). Only residents moved faster when their step started in a clearcut (Fig. 3D).

**Table 4:** Average habitat selection and movement rate coefficients for dispersers and residents at the landscape (RSF) and local scale (iSSA)

Category	Coefficient name	Resident					Dispersal				
		Estimate	CI lower	CI upper	Prop	Direction	Estimate	CI lower	CI upper	Prop	Direction
*Landscape scale (RSF)*											
Habitat selection	Bog	**− 0.638**	**− 0.801**	**− 0.476**	0.913	Avoided	**− 0.934**	**− 1.318**	**− 0.549**	0.933	Avoided
Habitat selection	Clearcut	0.001	**− **0.132	0.134	0.391	Indifferent	0.023	**− **0.263	0.310	0.333	Indifferent
Habitat selection	TRI	**0.049**	**0.032**	**0.066**	0.783	Selected	0.020	**− **0.011	0.051	0.533	Indifferent
Habitat selection	Dist.Water	0.044	**− **0.056	0.143	0.478	Indifferent	0.063	**− **0.094	0.220	0.533	Indifferent
Habitat selection	Dist.buildings	**0.324**	**0.219**	**0.430**	0.913	Avoided	**− **0.028	**− **0.114	0.059	0.400	Indifferent
Habitat selection	Dist.ForestryRoads	**− 0.042**	**− 0.051**	**− 0.032**	0.783	Selected	**− **0.008	**− **0.035	0.019	0.400	Indifferent
Habitat selection	Dist.PublicRoads	**0.295**	**0.146**	**0.443**	0.870	Avoided	0.002	**− **0.084	0.088	0.533	Indifferent
*Local scale (iSSA)*											
Habitat selection	Bog_end	**− 0.653**	**− 0.727**	**− 0.580**	0.935	Avoided	**− 0.959**	**− 1.198**	**− 0.719**	0.933	Avoided
Habitat selection	Clearcut_end	**− 0.423**	**− 0.520**	**− 0.326**	0.717	Avoided	**− 0.375**	**− 0.660**	**− 0.090**	0.600	Avoided
Habitat selection	TRI_end	**0.022**	**0.017**	**0.026**	0.913	Selected	0.005	**− **0.007	0.016	0.600	Indifferent
Habitat selection	Dist.Water_end	**0.119**	**0.096**	**0.142**	0.957	Avoided	**− **0.020	**− **0.099	0.059	0.333	Indifferent
Habitat selection	Dist.buildings_end	**0.060**	**0.021**	**0.100**	0.761	Avoided	**0.111**	**0.016**	**0.205**	0.800	Avoided
Habitat selection	Dist.ForestryRoads_end	**− 0.059**	**− 0.075**	**− 0.043**	0.804	Selected	**− **0.008	**− **0.054	0.038	0.467	Indifferent
Habitat selection	Dist.PublicRoads_end	**0.093**	**0.052**	**0.133**	0.826	Avoided	0.022	**− **0.074	0.118	0.800	Indifferent
Movement	SL	0.000	0.000	0.000	0.674	Indifferent	**0.000**	**− 0.001**	**0.000**	0.867	Faster
Movement	log (SL)	**− **0.010	**− **0.215	0.195	0.109	Indifferent	0.070	**− **0.277	0.416	0.867	Indifferent
Movement	log(SL)*clearcut_start	**0.120**	**0.076**	**0.163**	0.826	Faster	0.091	**− **0.033	0.215	0.867	Indifferent
Movement	log(SL)*Dist.buildings_start	0.008	**− **0.016	0.033	0.543	Indifferent	**− 0.032**	**− 0.058**	**− 0.007**	0.467	Faster
Movement	log(SL)*Dist.ForestryRoads_start	**− 0.071**	**− 0.082**	**− 0.061**	0.978	Faster	**− 0.070**	**− 0.106**	**− 0.033**	0.800	Faster
Movement	log(SL)*Dist.PublicRoads_start	**− 0.022**	**− 0.036**	**− 0.008**	0.717	Faster	**− 0.052**	**− 0.090**	**− 0.014**	0.667	Faster

Each bear-year was modelled separately before inverse-variance weighted linear models were run for both dispersers and residents. The proportion columns (‘prop’) give the proportion of the bear-years that had coefficient estimates in the same direction as the averaged coefficient (n = 15 for dispersers and n = 46 for residents). Bolded values indicate that the 95% confidence interval of the coefficients do not overlap zero. “Dist.” is an abbreviation for “distance to”

### Human infrastructure

At the population level and across scales, dispersers were indifferent to human infrastructure, except for buildings which they avoided at the local scale. Residents avoided buildings and public roads, and selected forestry roads at both local and landscape scale. Residents did not alter their movement rate closer to buildings, while dispersers moved faster closer to buildings (Table 4; Fig. 3C). Both dispersers and residents increased their movement rate closer to forestry roads and closer to public roads (Table 4).

Dispersers and residents moved 551 m/h and 599 m/h, respectively, faster when their step started on a forestry road compared with the movement rate when starting 500 m away from a forestry road (Fig. 3A). Similarly, dispersers and residents moved 141 m/h and 169 m/h, respectively, faster when their step started on a public road compared with the movement rate at 500 m away from a public road (Fig. 3B). In other words, the change in the movement speed for dispersers and residents was 3.26 and 3.54 times higher, respectively, on forestry roads compared with public roads.

## Discussion

We found evidence that human infrastructure is important for describing habitat selection and movement (P1 in support of H1) regardless of life stage (i.e. resident or disperser) at both the local and landscape scale, as models containing human infrastructure performed best. However, at the landscape scale, dispersers appeared indifferent towards most landscape features, including human infrastructure (Table 4 and Additional file 2: Fig. S2). Dispersers were more often indifferent towards human infrastructure at both scales (P2 in support of H2) and did not show the same avoidance patterns of human infrastructure as residents. Dispersers were less sensitive towards human infrastructure on the landscape scale than the local scale, however, this did not apply to residents (P3 partial support of H3). Furthermore, dispersers more often exhibited indifference to human infrastructure in both habitat selection and movement rates.

Human infrastructure appears important for both dispersers and resident at the local scale and the landscape scale (H1). At the local scale, dispersers and residents avoided buildings, and dispersers moved faster closer to buildings suggesting they are perceived as risky habitat. The lack of increased movement rate closer to buildings by residents might be due to residents generally being farther away from buildings. Both dispersers and residents moved faster when closer to public and forestry roads. Whether a road facilitates or impedes movement is likely dependent on the traffic volume [66]. This was presumably the case in our study area, as residents appeared to treat public roads as risky habitat (avoiding and moving faster), whereas they used the smaller forestry roads for travel (selecting and moving faster). The forestry roads are assumed to generally have low levels of human activity, and even lower at night when bears moved more (Additional file 2: Fig. S1). Mortality risk for bears along all roads during our study period was likely low, as traffic accidents do not account for a large proportion of mortality [< 2%; 67] and our study ended before the onset of the hunting season, after which roads have an additive effect on hunting success [68]. Movement facilitation in relation to linear features, such as roads, has also been observed for brown bears in other areas [60], while treating public roads as risky has been reported for other species [69, 70]. Dispersers were indifferent in terms of habitat selection for either road type and traveled faster near both, indicating that public and forestry roads may potentially serve as risky features [71] or as facilitators of movement [72] during dispersal. This indifference might be explained by the dispersers lack of information on where to find roads, or large variation in the population of dispersers where some avoid roads while others may select for them.

Although the best models at the landscape scale contained human infrastructure for both life stages (support of H1), dispersers were mainly indifferent to human infrastructure. They also used habitat closer to human infrastructure compared with residents. This is in support of H2, i.e. dispersers are either more naïve or risk-tolerant to human infrastructure. In contrast, residents were sensitive to human infrastructure and avoided both public roads and buildings at both availability scales. Hence, we suggest that naivety or risk-tolerance plays a prominent role in the behavior of dispersers when navigating novel landscapes. Dispersal is often considered risky due to movement through novel landscapes [73], and human-derived risks can have an additive effect beyond what is ‘normally expected’ by animals when weighing the decision whether to disperse and where to go [13]. In contrast, the assumption that residents are more familiar with their home ranges and dispersers must navigate novel terrain, is supported by the residents’ avoidance of potential human-derived risk at both local and landscape scales.

Alternatively, areas with lower human mortality risk might not be preferred by dispersers, due to increased intraspecific mortality risk or competition from larger, adult males [74, 75], which would effectively sandwich dispersing males between two sources of mortality risk [sensu] [76], i.e. humans and conspecifics. This is also consistent with our core covariate findings, i.e., residents selected for rugged terrain, while dispersers did not. Similarly, clearcuts and bogs are open habitats in which bears have less cover from human detection [77]. Such habitats were avoided by both dispersers and residents at the local scale, indicating a similar avoidance of potential human-derived risk in these open or semi-open habitat types. Dispersing males in other large carnivores, such as African lions (*Panthera leo*) [8], African wild dogs (*Lycaon pictus*) [78] and gray wolves [7], depict a similar pattern with weaker or no avoidance of human infrastructure. Lack of avoidance of human infrastructure may be driven by the attempt to avoid of larger males. However, it could also be explained by the dispersing individual’s inability to detect these features at relevant distances. Similarly, the lack of selection for landscape features preferred by older males, e.g. the lack of selection for rugged terrain by dispersers, may also be explained by the dispersing males not knowing where to find these features when moving through the landscape. This is supported by dispersers not selecting for any features on any of the scales. Alternatively, adult male habitat selection may emerge from learning, i.e. adjusting from being a dependent with its mother to that of an adult male.

Our selection of covariates might be more applicable toward residents, as it is based on the literature where more information is available for residents compared to dispersers. However, dispersers likely consider and respond to similar habitat features as residents, even though responses may differ. This is supported by our results, as the full model was best for both life stages and candidate models had identical sorting based on the mean ∆AIC and at both spatial scales (Table 4). We attribute the higher proportion of non-significant coefficients in the disperser models to higher individual variation among dispersers. The more risk-tolerant or naïve behavior in dispersers [7, 8] combined with a high level of individual variation may be important to maintain structural and functional connectivity between populations, as bolder or more risk-tolerant individuals [79] may disperse more effectively through human-modified landscapes. At the same time, this also implies that areas important for connectivity with a high human footprint may experience higher levels of human-bear conflict with implications for functional connectivity, unless carefully designed mitigation strategies are adopted [80].

We found partial support of H3, that the scale of availability influences how sensitive bears are towards human infrastructure, as only dispersers were more selective at the local scale compared with the landscape scale (P3). This is in contrast to what has been observed in pronghorn antelopes (*Antilocapra americana*) which showed stronger avoidance at the landscape scale than the local scale during migration [81], illustrating the existence of different strategies for which spatial scale animals avoid human infrastructure. Furthermore, dispersing brown bear males more often treated human infrastructure indifferently in habitat selection and movement rates at the local scale (Table 4). In our study area, bears are intensively hunted annually [upwards of 10% of the population; 82], putting them into contact with human-derived risk virtually everywhere in the study area, and bears modify their behavior to minimize human predation risk [17, 48, 83, 84]. Hence, resident bears likely select areas where human impact is low [35] and further reduce potential encounter rates with humans through behavioral changes on local spatial scales and temporally [28, 48]. As dispersers were more exposed to human infrastructure, they are potentially also more exposed to higher human predation risk. Indeed, hunting mortality of males compared to females appears to be higher at the onset of dispersal in this bear population [68, 85], which could partially be due to male-biased dispersal [41], increased risk-taking or tolerance during dispersal, and the inability of dispersing males to adequately recognize and adjust to novel human-derived mortality risk.

## Conclusions

Our study highlights that life stage can influence how individuals respond to human landscape features across scales. The key to attaining reliable connectivity models is the recognition that animal dispersal decisions and movement patterns are life stage dependent. Our findings suggest that risk-tolerant or naïve dispersers might use movement pathways that more risk-averse or habitat-familiar residents would avoid. As a result, landscape resistance or connectivity maps derived from dispersal movement data may provide more numerous as well as more realistic pathways than those derived from resident movement data alone [86–88]. At the same time, the increased risk-tolerance or naivety in dispersers has the potential to exacerbate human-bear conflicts in important connectivity areas. Hence, identifying these areas early on from connectivity maps can help to mitigate human-bear conflict. The differences in scale-specific decisions between dispersers and residents provide the foundation for understanding functional connectivity of a population, in which animals disperse and establish a home range for subsequent reproduction [89]. Attaining individual-based dispersal data on large carnivores is costly but informative, particularly in human-dominated systems where coexistence has been touted as not only possible, but imperative on some level for large carnivore persistence [90–92].

## Supplementary Information

**Additional file 1: Supplement S1.** Description for hidden Markov model (HMM) to define the transient period of dispersal.

**Additional file 2: Figure S1**. The number of GPS locations at given hours of the day prior to bed removal (A) and after bed removal (B). **Figure S2**. Coefficient plots from resource selection functions (A) and the integrated step selection analysis (B). Yellow color indicate dispersing males and blue color indicate resident males. **Table S1**. The mean availability coefficient for the weighted linear models calculating the population estimates. Significant coefficients are indicated in bold and indicate a functional response, i.e. that the effect of the covariate variates with the availability. “D2.” is an abbreviation for “distance to”.

## Data Availability

The datasets used and analysed during the current study are available from the corresponding author on reasonable request.
