# Supplementary material for: Movement and habitat selection of a large carnivore in response to human infrastructure differs by life stage
Source: Mov Ecol. 2022 Nov 29;10:52. doi: 10.1186/s40462-022-00349-y (PMC9706841; doi:10.1186/s40462-022-00349-y)
Supplement: Supplementary file 1 — Additional file 1: Supplement S1. Description for hidden Markov model (HMM) to define the transient period of dispersal. [file 40462_2022_349_MOESM1_ESM.pdf]

Supplement S1

Human impact on life stage dependent movement in male brown bear (Ursus arctos)

Step-by-step documentation for HMM fitting procedure

In this supplement, we go step-by-step through the fitting procedure of hidden Markov models for an individual male bear, W0612, and the selection of dates for the dispersal process.

Load required packages:

```
library(moveHMM)
library(doBy)
library(ggplot2)
library(plyr)
library(plotly)
```

Importing and prepping the data

Import geolocations* from one male bear (W0612)

```
W0612 <- readRDS("C:/Users/jeh/Documents/USN-PhD/Analysis/Bear_Movement/Trajectory_Splitting/W0612_anon

# subset to active season, 01 April - 01 October

W0612 <- subset(W0612, format(GMT.date,'%m%d')>='04-01' & format(GMT.date, '%m%d')
                <= '10-02')
```

Create a 'move' object from geolocations. This will calculate distance traveled between each step as well as turning angles of movement.

```
W0612Move <- prepData(W0612, type = "UTM", coordNames = c("x1", "y1"))
```

We keep only steps that are separated by an hour. This prevents unrealistic step lengths and turning angles from being included in the modeling procedure.

```
# copy to a new object called "W0612Reg"

W0612Reg <- W0612Move

# get time differences between each step

W0612Reg$diffHour <- difftime(W0612Move$GMT.date[1:length(W0612Move$GMT.date)] ,
```

```
                              W0612Move$GMT.date[2:length(W0612Move$GMT.date)])

# keep only times that are separated by an hour

W0612Reg <- W0612Reg[W0612Reg$diffHour >= -1 & W0612Reg$diffHour < 2, ]

# remove column for time difference

W0612Reg$diffHour <- NULL
```

Fitting HMMs

In the following section, we fit three models with two behavioral states, representing short and long movement patterns. Each set of initial parameters are different, which increases the chance of finding the global maximum (citation).

Fitting a hidden Markov model to movement data requires four starting parameters specified for n behavioral states. The first parameter is mean step length distance, mu. The second is the standard deviation for each mean, sigma. The third parameter is the mean turning angle, angleMean. The final parameter is kappa, concentration of the turning angle.

The first set of initial parameters were chosen by looking at summaries of the step lengths and turning angles, histograms and density plots. The second set of initial parameters were "wider" (smaller/larger) values than the first set. The initial parameters for the third model were the estimated parameters from the previous model.

There is no exact science behind the selection of initial parameters, but [practical guidance] (https://cran.r-project.org/web/packages/moveHMM/vignettes/moveHMM-starting-values.pdf) is available.

The model specification is as follows:

fitHMM(move_object, number of behavior states, initial parameters, theoretical distribution for step lengths, theoretical distribution for turning angles)

We selected the gamma distribution for the step lengths and the vonMises distribution for the turning angles.

```
# first set of initial parameters

mu2a <- c(10, 1000)
sigma2a <- c(20, 2000)
angleMean2a <- c(pi, 0.005)
kappa2a <- c(0.2, 2)
stepPar2a <- c(mu2a, sigma2a)
anglePar2a <- c(angleMean2a, kappa2a)

# fit the first 2-state HMM

W0612_2a <- fitHMM(W0612Reg, nbStates = 2, stepPar0 = stepPar2a, anglePar0 = anglePar2a,
                   stepDist = "gamma", angleDist = "vm")
W0612_2a
```

```
## Value of the maximum log-likelihood: -26234.64
##
## Step length parameters:
## ----------------------
```

```
##        state 1   state 2
## mean 17.00918 1042.0373
## sd   14.02674  924.3737
##
## Turning angle parameters:
## ------------------------
##                  state 1     state 2
## mean          -2.9787585 -0.01945528
## concentration  0.4975304  0.93201121
##
## Regression coeffs for the transition probabilities:
## --------------------------------------------------
##              1 -> 2    2 -> 1
## intercept -1.281184 -1.476296
##
## Transition probability matrix:
## -----------------------------
##           [,1]      [,2]
## [1,] 0.7826513 0.2173487
## [2,] 0.1859875 0.8140125
##
## Initial distribution:
## --------------------
## [1] 1.154684e-06 9.999988e-01
```

```
# calculate confidence intervals and plot the fitted model results

W0612_2_a <- CI(W0612_2a)
plot(W0612_2a, plotCI = TRUE)
```

```
## Decoding states sequence... DONE
```

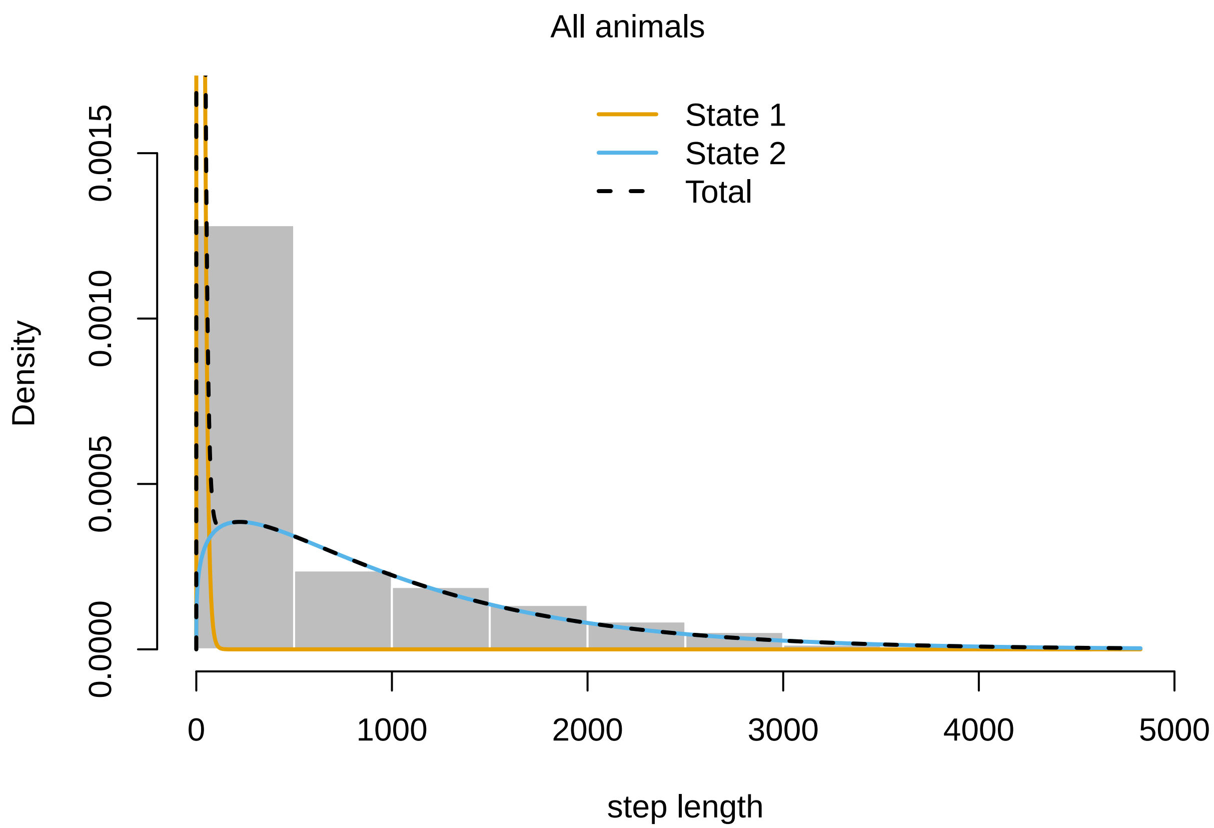
All animals
State 1
State 2
Total
Density
0.0000
0.0005
0.0010
0.0015
0
1000
2000
3000
4000
5000
step length

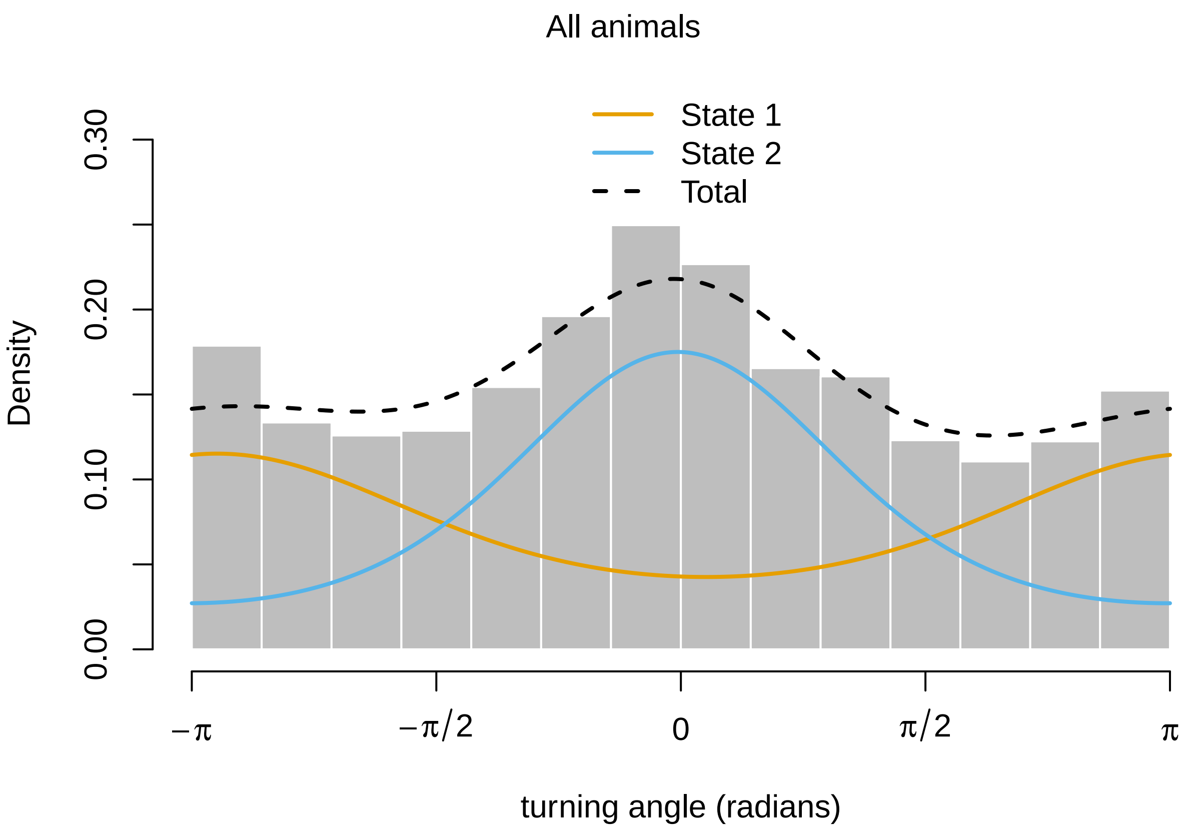
All animals
State 1
State 2
Total
Density
0.00
0.10
0.20
0.30
−π
−π/2
0
π/2
π
turning angle (radians)

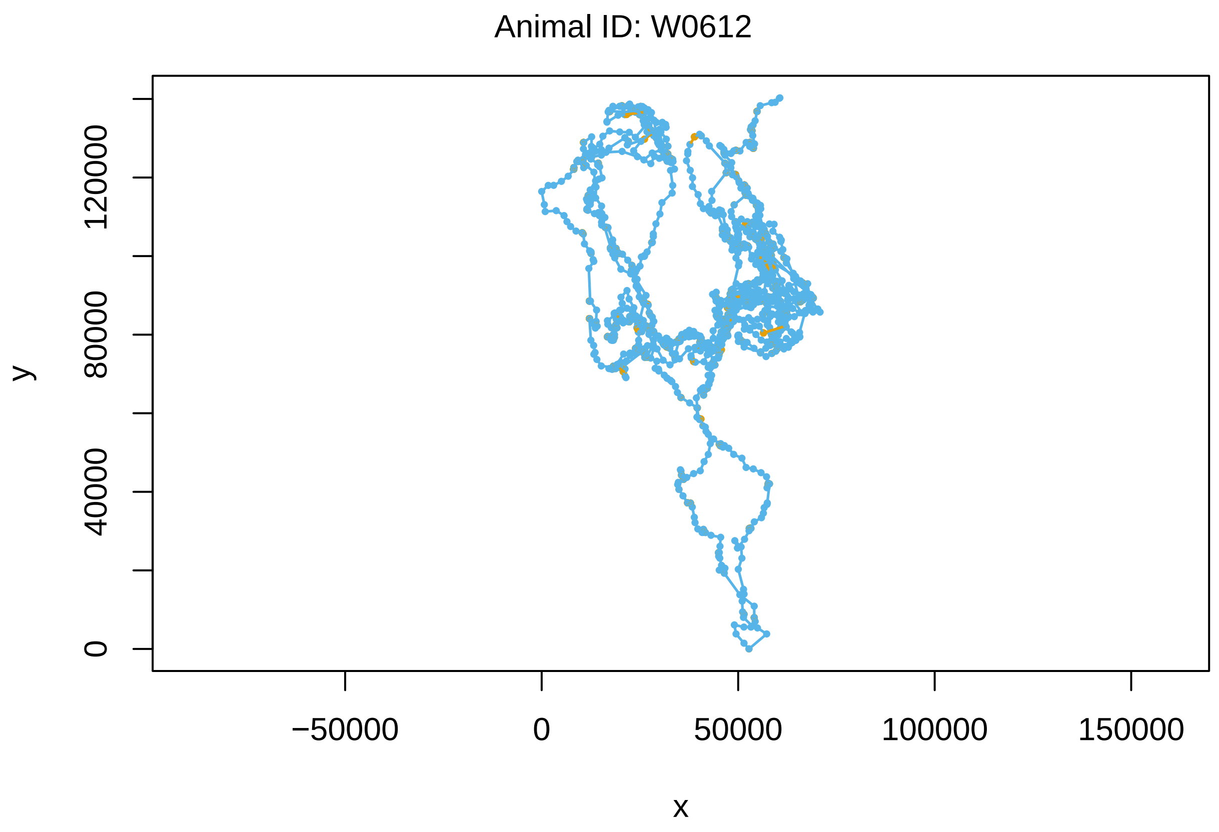

The Viterbi algorithm (Zucchini and MacDonald 2009) assigns probable behavioral states to each step. The plotStates function returns a plow showing the probability of being in a particular state at each observation index location (step).

```
head(viterbi(W0612_2a), n = 20)
```

```
##  [1] 2 2 2 2 2 1 1 1 1 1 1 1 1 1 1 1 2 2 2 2
```

```
plotStates(W0612_2a)
```

```
## Decoding states sequence... DONE
## Computing states probabilities... DONE
```

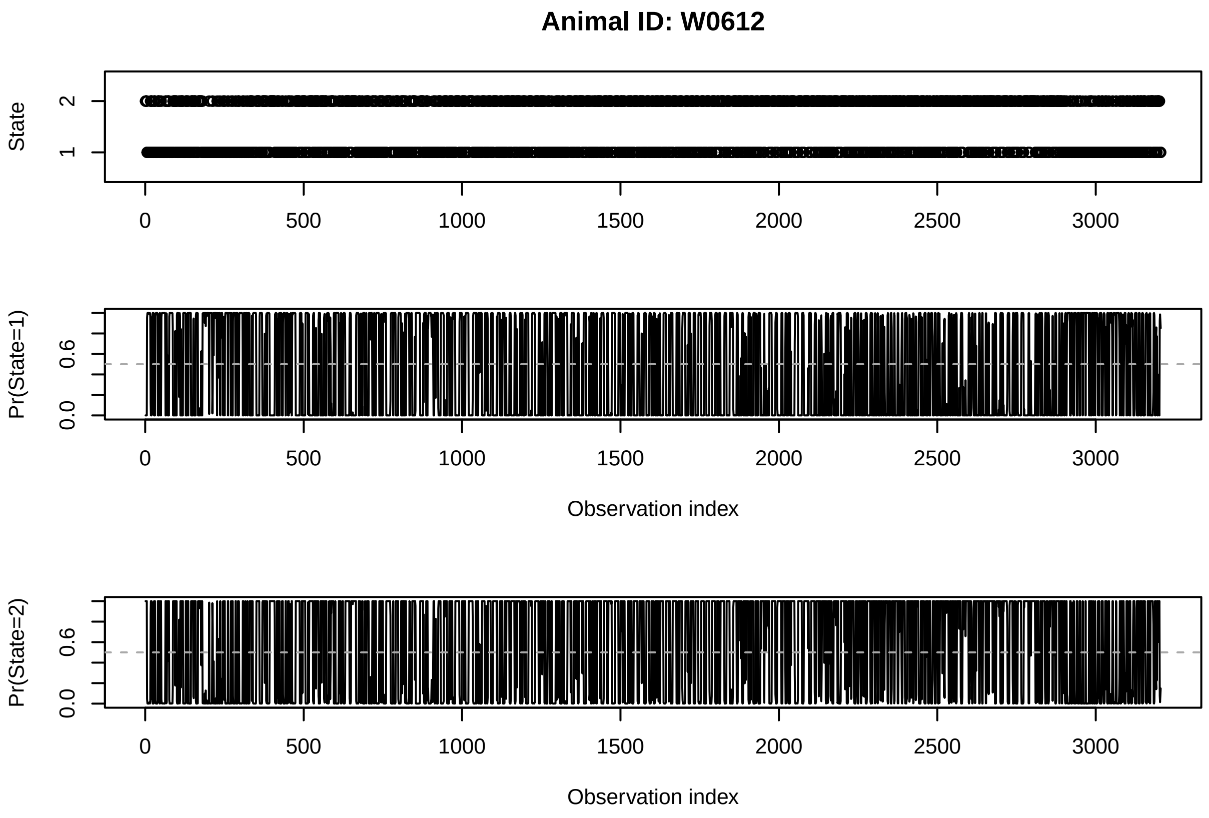

The final step of the process is to look at the residuals to assess the fit of the model to the data. Plotting the model object directly will show the time series, qq-plots, and sample ACF functions of the pseudo-residuals for steps and turning angles. We also produced histograms of the pseudo-residuals for step lengths and turning angles.

```
plotPR(W0612_2a)
```

```
## Computing pseudo-residuals... DONE
```

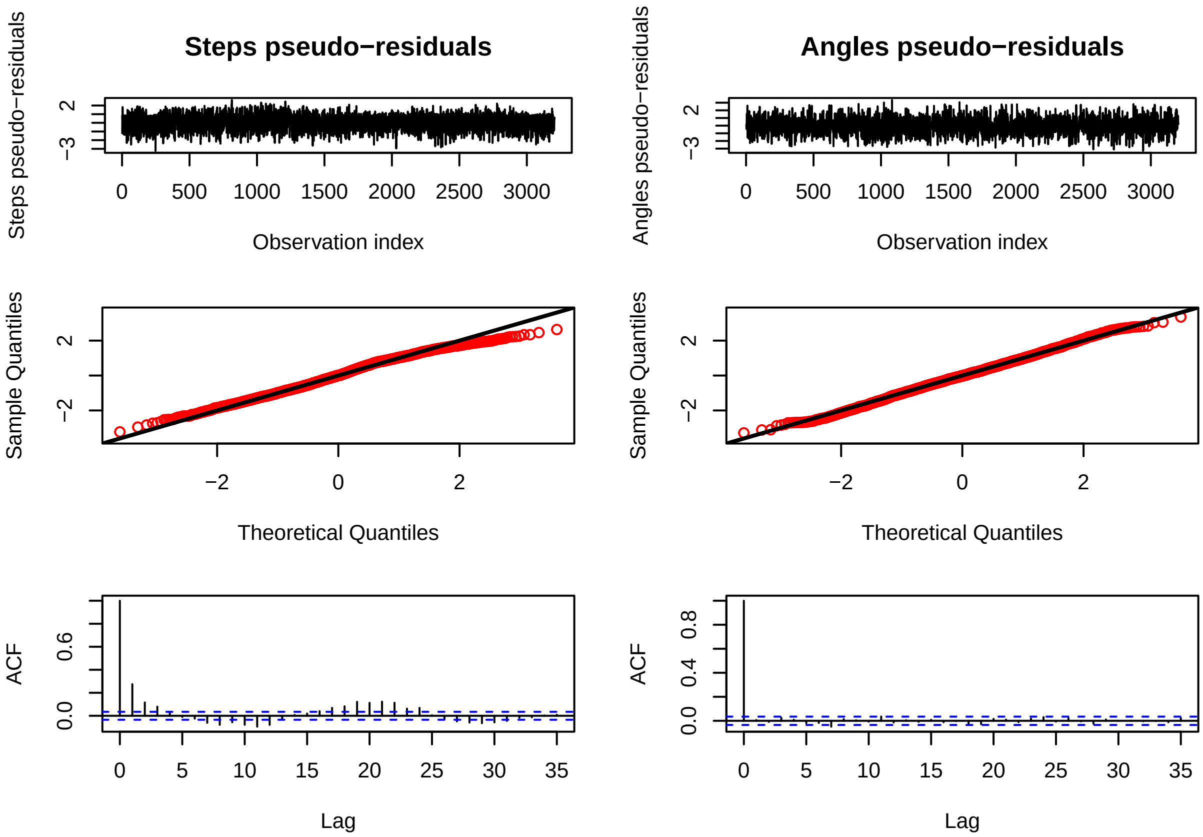


```
W0612_2a_res <- pseudoRes(W0612_2a)
hist(W0612_2a_res$stepRes)
```

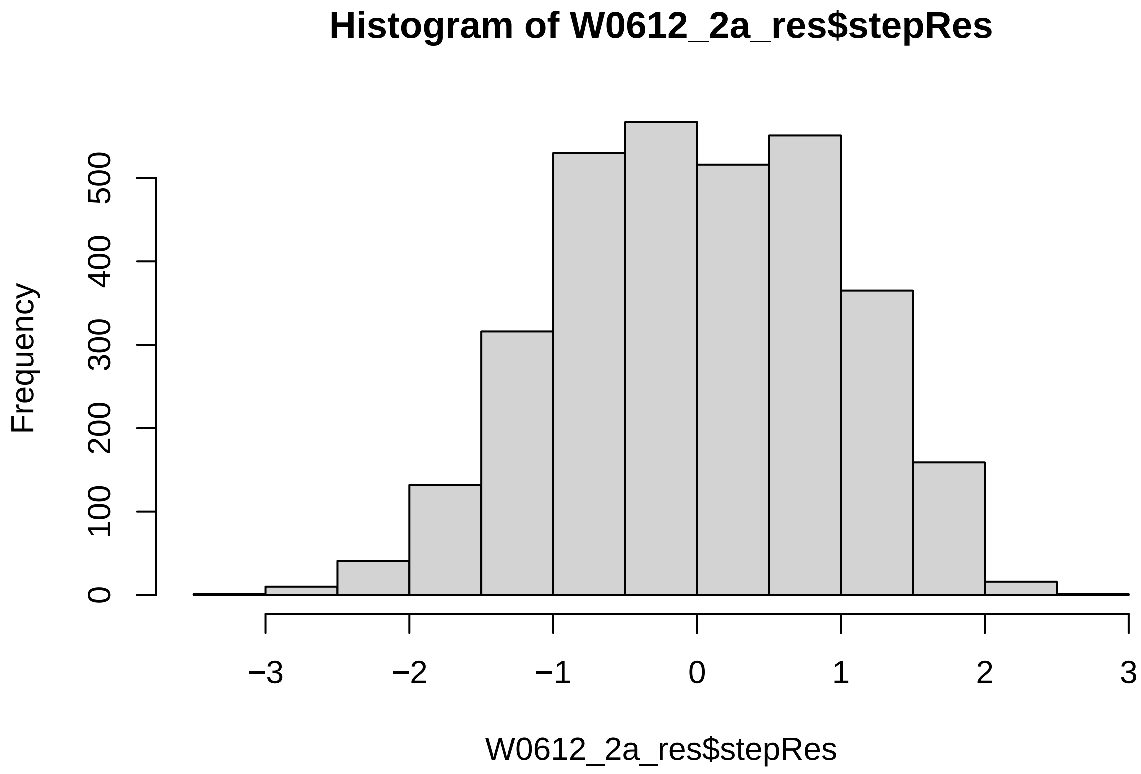


```
hist(W0612_2a_res$angleRes)
```

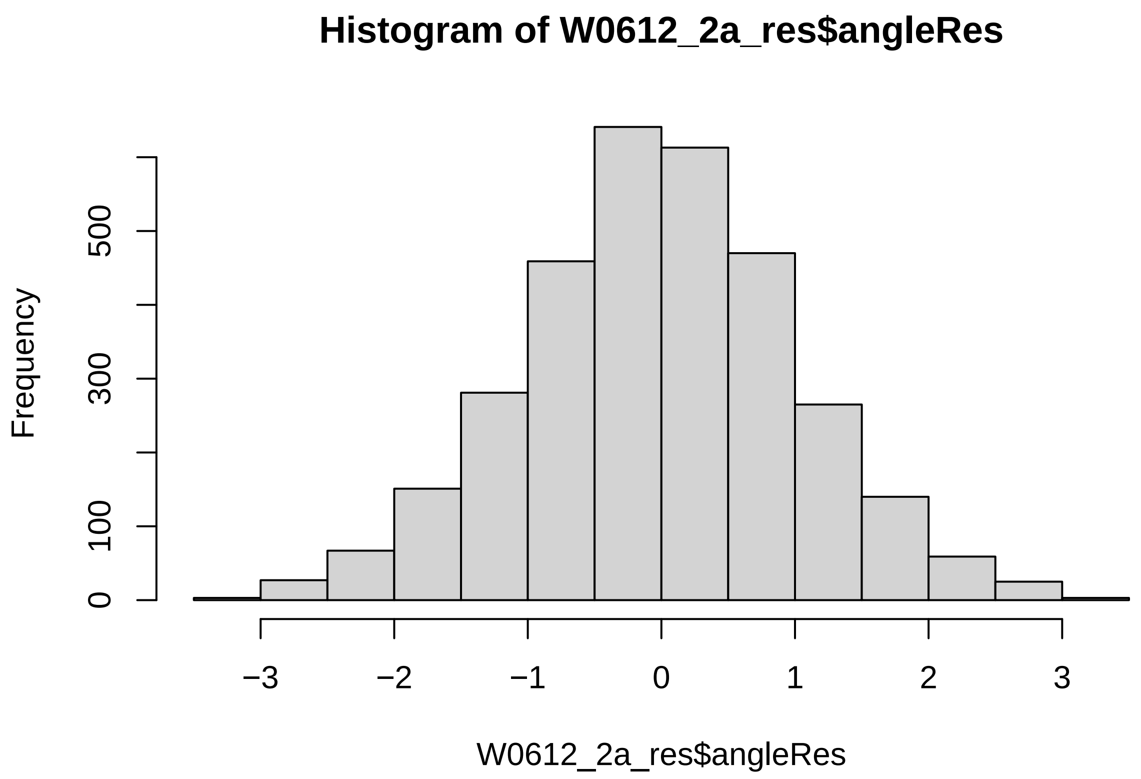

We fit the remaining two models with different initial parameters.

```
# second set of parameters (wider values)

mu2b <- c(5, 2000)
sigma2b <- c(8, 1000)
angleMean2b <- c(pi, -0.005)
kappa2b <- c(0.1, 1)
stepPar2b <- c(mu2b, sigma2b)
anglePar2b <- c(angleMean2b, kappa2b)


# fit the second 2-state HMM

W0612_2b <- fitHMM(W0612Reg, nbStates = 2, stepPar0 = stepPar2b, anglePar0 = anglePar2b,
                   stepDist = "gamma", angleDist = "vm")
W0612_2b
```

```
## Value of the maximum log-likelihood: -26234.64
##
## Step length parameters:
## ----------------------
##       state 1   state 2
## mean 17.00917 1042.0356
## sd   14.02674  924.3724
##
```

```
## Turning angle parameters:
## -------------------------
##                    state 1     state 2
## mean           -2.9787801 -0.01945568
## concentration  0.4975309  0.93201136
##
## Regression coeffs for the transition probabilities:
## ----------------------------------------------------
##               1 -> 2    2 -> 1
## intercept -1.281185 -1.476297
##
## Transition probability matrix:
## ------------------------------
##           [,1]      [,2]
## [1,] 0.7826515 0.2173485
## [2,] 0.1859874 0.8140126
##
## Initial distribution:
## ---------------------
## [1] 4.445916e-07 9.999996e-01
```

```
# third set of parameters (estimated parameters from previous model)

mu2c <- c(17, 1042)
sigma2c <- c(14, 924)
angleMean2c <- c(-2.98, -0.02)
kappa2c <- c(0.5, 0.93)
stepPar2c <- c(mu2c, sigma2c)
anglePar2c <- c(angleMean2c, kappa2c)


# fit the third 2-state HMM

W0612_2c <- fitHMM(W0612Reg, nbStates = 2, stepPar0 = stepPar2c, anglePar0 = anglePar2c,
                   stepDist = "gamma", angleDist = "vm")
W0612_2c
```

```
## Value of the maximum log-likelihood: -26234.64
##
## Step length parameters:
## ----------------------
##       state 1   state 2
## mean 17.00917 1042.0380
## sd   14.02674  924.3724
##
## Turning angle parameters:
## -------------------------
##                   state 1    state 2
## mean           -2.978775 -0.0194565
## concentration  0.497498  0.9320255
##
## Regression coeffs for the transition probabilities:
## ----------------------------------------------------
##               1 -> 2    2 -> 1
```

```
## intercept -1.281186 -1.476288
##
## Transition probability matrix:
## -----------------------------
##           [,1]      [,2]
## [1,] 0.7826515 0.2173485
## [2,] 0.1859887 0.8140113
##
## Initial distribution:
## --------------------
## [1] 8.609635e-06 9.999914e-01
```

Results of the 2-state modeling indicated a poor fit of step lengths in the model. We continue with fitting a 3-state model with the following behaviors: resting, foraging/short-distance movement, and directed, long-distance traveling.

Fitting of the 3-state HMM is the same as for a 2-state except that we have three values for each parameter that represent the behavioral states. Initial parameter values for the 3-state HMMs were chosen as follows:

1. Educated guess based on step length and turning angle summaries.
2. Creating three equal bins for the distributions.
3. From a combination histogram-density plot in ggplot2.
4. The estimated parameters from the previous model.

```
# initial parameters for 3-state model

mu3a <- c(4, 200, 3500)
sigma3a <- c(5, 300, 1500)
angleMean3a <- c(pi, 0.005, 0.05)
kappa3a <- c(0.1, 0.5, 1)
stepPar3a <- c(mu3a, sigma3a)
anglePar3a <- c(angleMean3a, kappa3a)


# fit the first 3-state HMM

W0612_3a <- fitHMM(W0612Reg, nbStates = 3, stepPar0 = stepPar3a, anglePar0 = anglePar3a,
                   stepDist = "gamma", angleDist = "vm")
W0612_3a
```

```
## Value of the maximum log-likelihood: -25864.43
##
## Step length parameters:
## ----------------------
##       state 1  state 2   state 3
## mean 13.87421 388.2844 1604.8023
## sd   10.06471 396.6112  749.0172
##
## Turning angle parameters:
## ------------------------
##                  state 1    state 2      state 3
## mean          -2.9698675 -0.1243276 -0.005814789
## concentration  0.5097004  0.2057111  1.868910436
```

```
##
## Regression coeffs for the transition probabilities:
## ---------------------------------------------------
##                1 -> 2    1 -> 3     2 -> 1     2 -> 3    3 -> 1    3 -> 2
## intercept -1.289126 -3.098643 -0.6737897 -0.9160029 -2.426462 -1.161481
##
## Transition probability matrix:
## -----------------------------
##              [,1]      [,2]       [,3]
## [1,] 0.75721904 0.2086225 0.03415843
## [2,] 0.26691251 0.5235909 0.20949663
## [3,] 0.06304459 0.2233685 0.71358691
##
## Initial distribution:
## --------------------
## [1] 1.873944e-06 9.999981e-01 3.746110e-09
```

```
# calculate confidence intervals and plot the fitted model results

W0612_3a_CI <- CI(W0612_3a)

plot(W0612_3a, plotCI = TRUE)
```

```
## Decoding states sequence... DONE
```

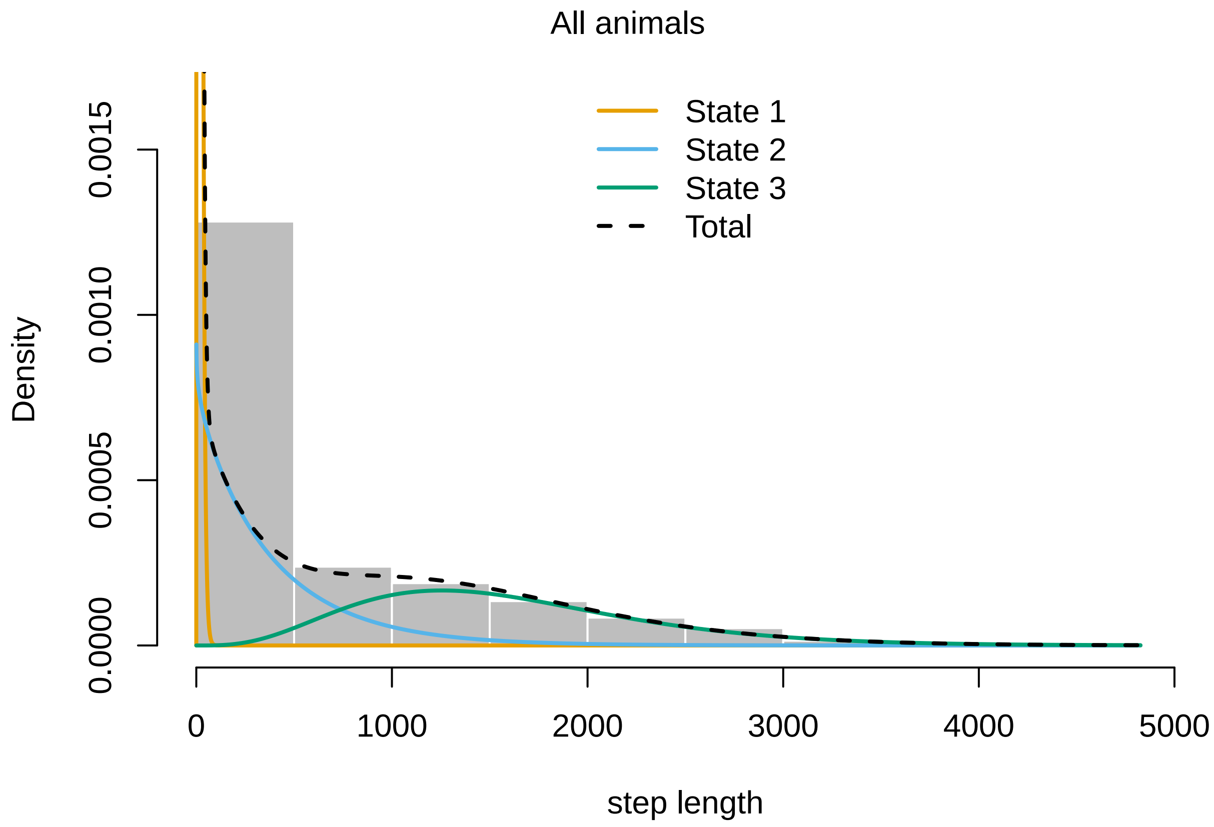

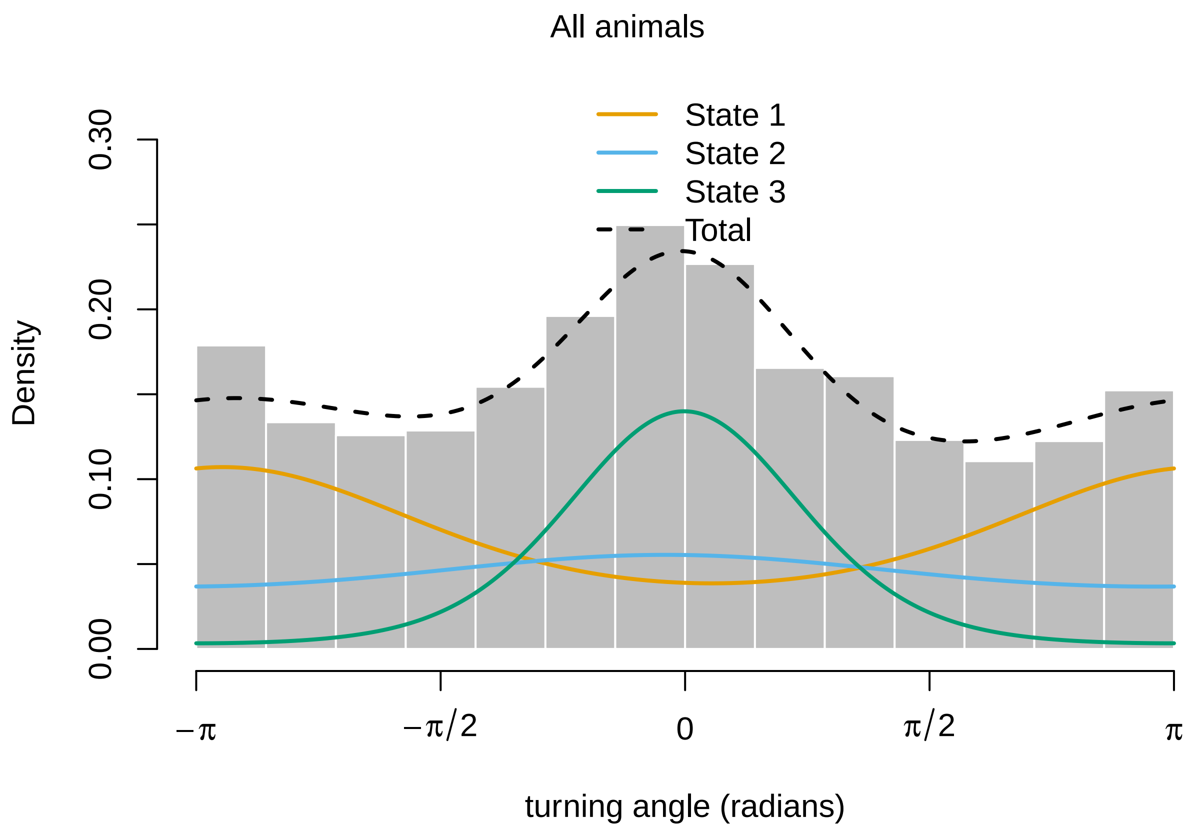
All animals
State 1
State 2
State 3
Total
Density
0.00
0.10
0.20
0.30
−π
−π/2
0
π/2
π
turning angle (radians)

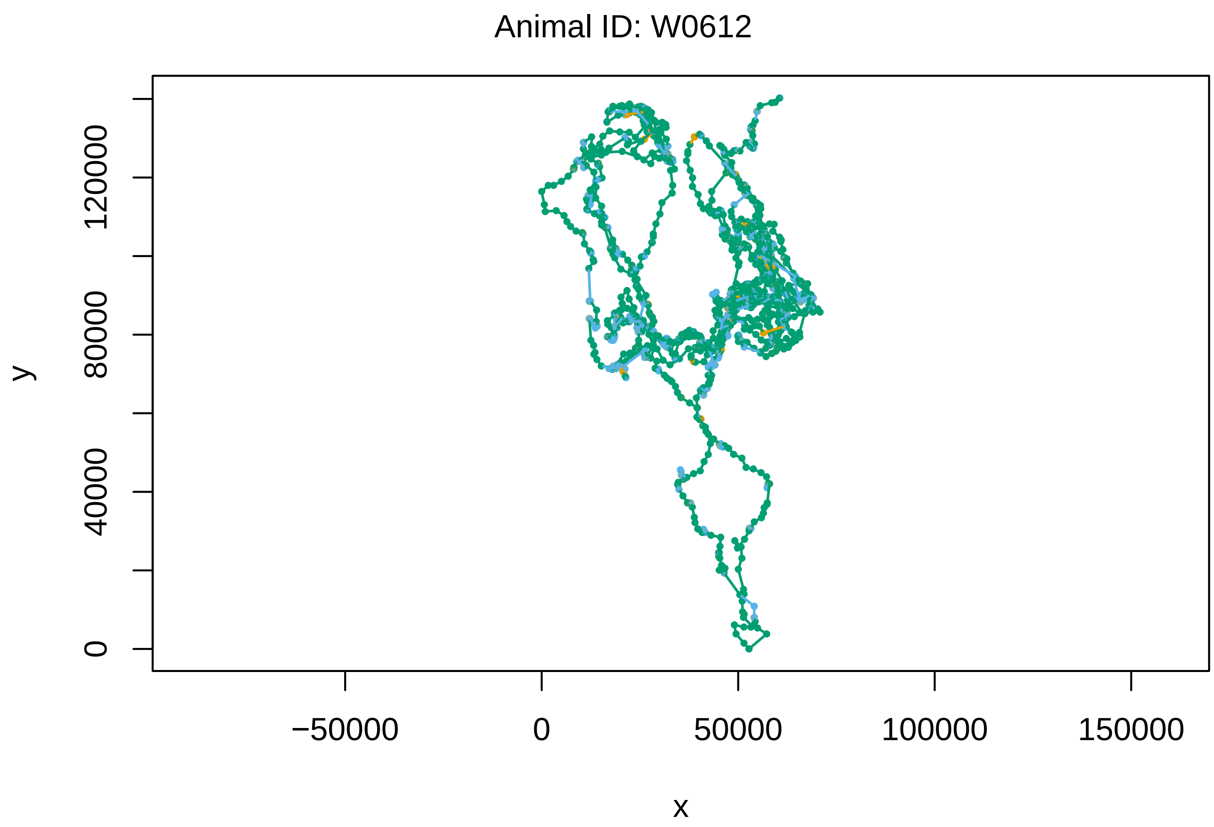

As before, we use the Viterbi algorithm to classify the states for each step.

```
head(viterbi(W0612_3a), n = 20)
```

```
##  [1] 2 3 3 3 3 2 1 1 1 1 1 1 1 1 1 1 2 3 3 3
```

```
plotStates(W0612_3a)
```

```
## Decoding states sequence... DONE
## Computing states probabilities... DONE
```

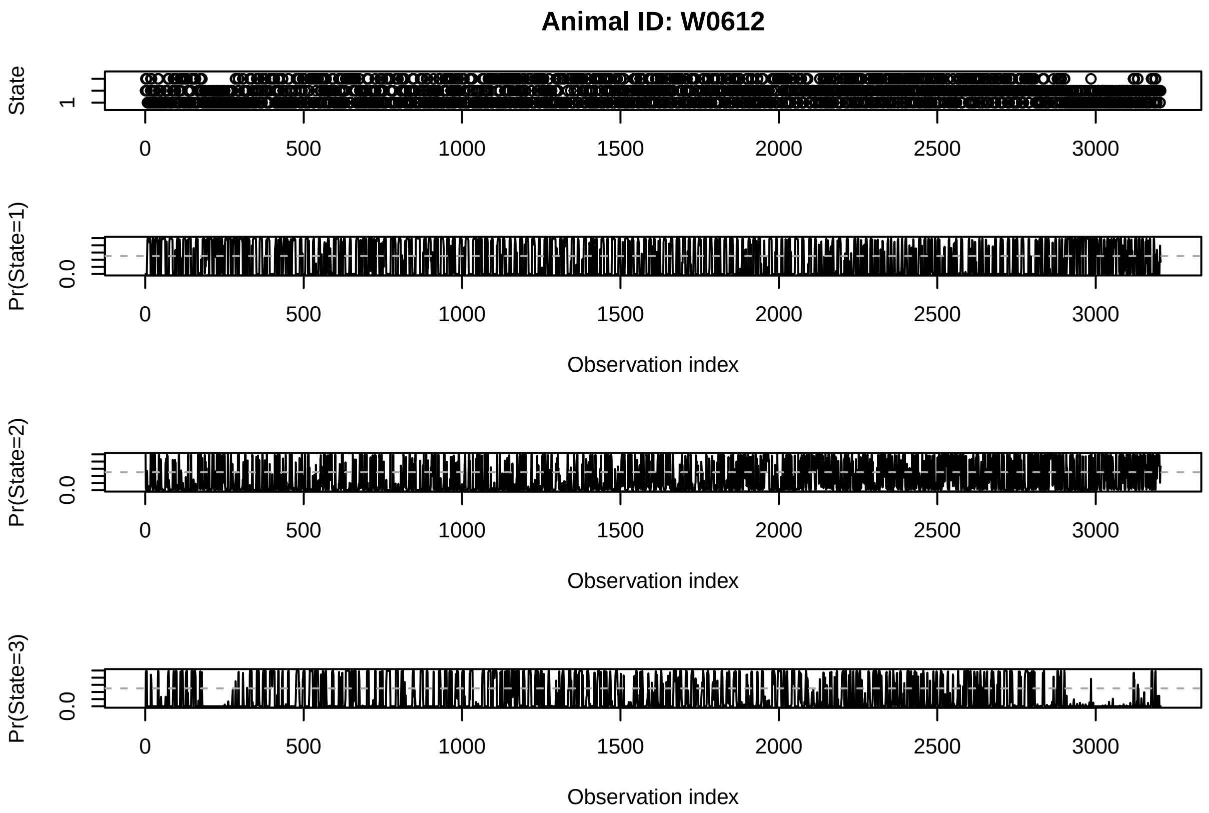

Inspect the pseudo-residuals for model fit.

```
plotPR(W0612_3a)
```

```
## Computing pseudo-residuals... DONE
```

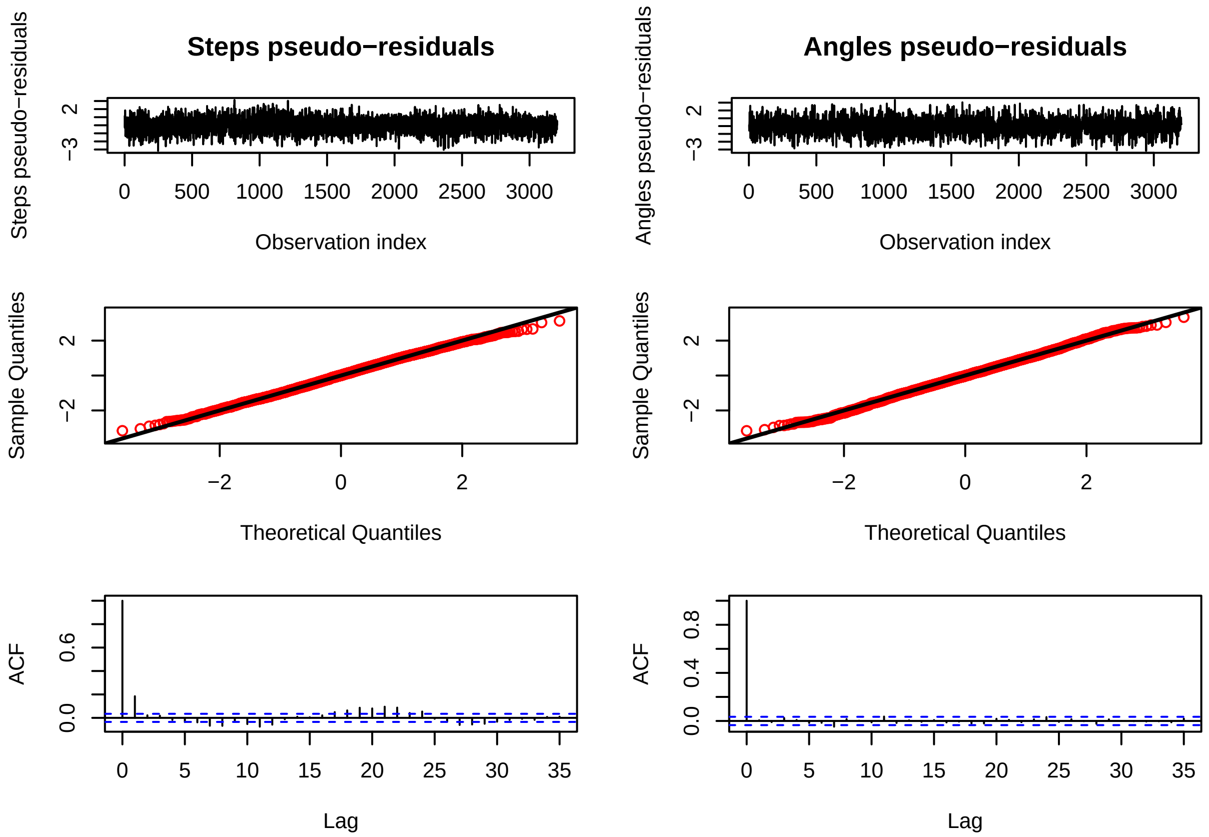


```
W0612_3a_res <- pseudoRes(W0612_3a)
hist(W0612_3a_res$stepRes)
```

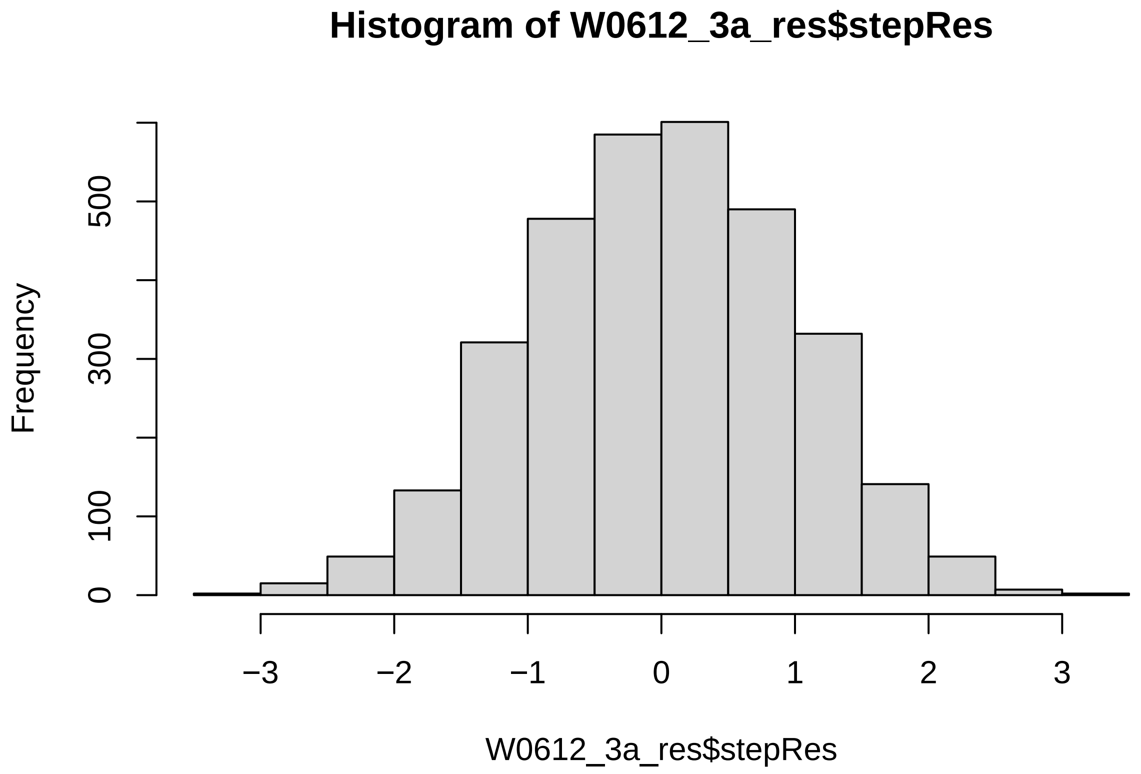


```
hist(W0612_3a_res$angleRes)
```

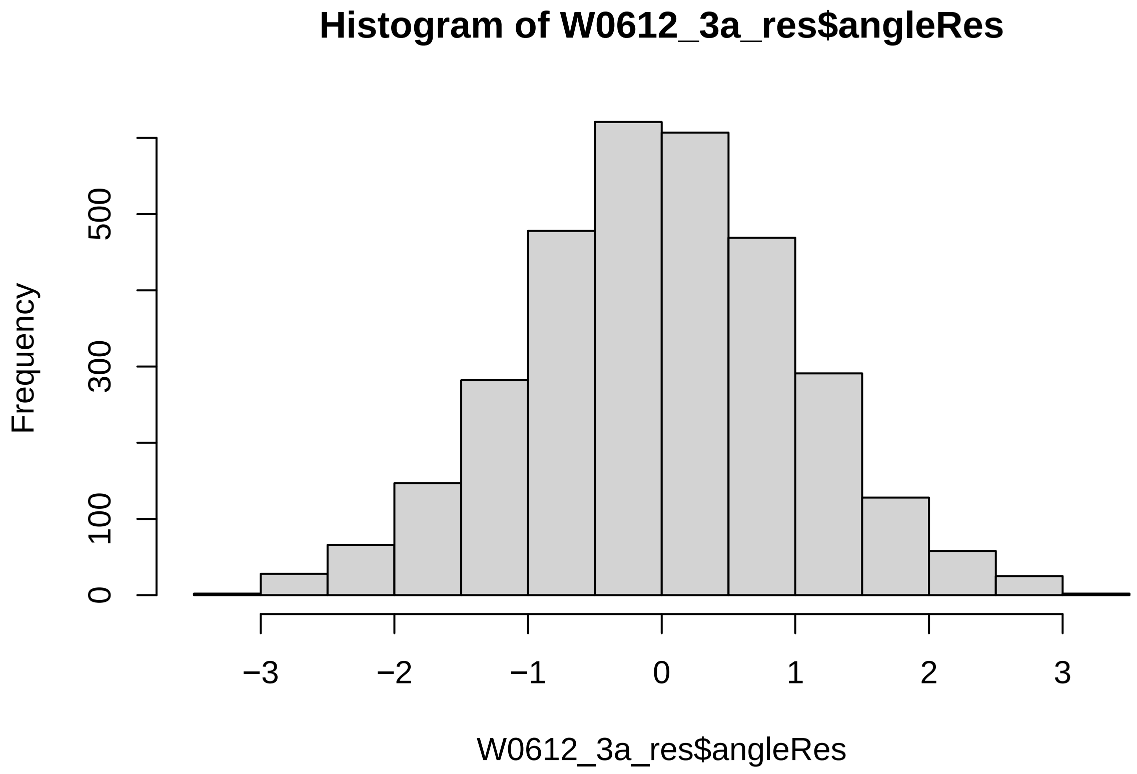

Fit the remaining three 3-state HMMs.

```
# second set of parameters (from equal binning)

mu3b <- c(10, 300, 2000)
sigma3b <- c(5, 150, 750)
angleMean3b <- c(pi,-0.005974, 1.57)
kappa3b <- c(1, 1, 1)
stepPar3b <- c(mu3b, sigma3b)
anglePar3b <- c(angleMean3b, kappa3b)

# fit the second 3-state HMM

W0612_3b <- fitHMM(W0612Reg, nbStates = 3, stepPar0 = stepPar3b, anglePar0 = anglePar3b,
                   stepDist = "gamma", angleDist = "vm")
W0612_3b
```

```
## Value of the maximum log-likelihood: -25864.43
##
## Step length parameters:
## ----------------------
##       state 1  state 2  state 3
## mean 13.87423 388.2872 1604.803
## sd   10.06474 396.6137  749.017
##
## Turning angle parameters:
```

```
## -------------------------
##                  state 1    state 2      state 3
## mean           -2.969865 -0.1243188 -0.005815661
## concentration  0.509698  0.2057129  1.868911138
##
## Regression coeffs for the transition probabilities:
## --------------------------------------------------
##              1 -> 2    1 -> 3     2 -> 1     2 -> 3    3 -> 1    3 -> 2
## intercept -1.28913 -3.098652 -0.6737915 -0.9160046 -2.426423 -1.161484
##
## Transition probability matrix:
## -----------------------------
##            [,1]      [,2]       [,3]
## [1,] 0.7572199 0.2086219 0.03415817
## [2,] 0.2669122 0.5235913 0.20949646
## [3,] 0.0630469 0.2233675 0.71358556
##
## Initial distribution:
## ---------------------
## [1] 5.665121e-07 9.999994e-01 6.391494e-10
```

```
# third set of parameters (from histogram-density plot in ggplot2)

mu3c <- c(25, 500, 1500)
sigma3c <- c(30, 300, 750)
angleMean3c <- c(pi,-0.005974, 1.57)
kappa3c <- c(0.5, 0.75, 1)
stepPar3c <- c(mu3c, sigma3c)
anglePar3c <- c(angleMean3c, kappa3c)

# fit the third 3-state HMM

W0612_3c <- fitHMM(W0612Reg, nbStates = 3, stepPar0 = stepPar3c, anglePar0 = anglePar3c,
                   stepDist = "gamma", angleDist = "vm")
W0612_3c
```

```
## Value of the maximum log-likelihood: -25864.43
##
## Step length parameters:
## ----------------------
##       state 1  state 2   state 3
## mean 13.87423 388.2859 1604.8017
## sd   10.06474 396.6122  749.0182
##
## Turning angle parameters:
## -------------------------
##                   state 1    state 2      state 3
## mean           -2.9698641 -0.1243471 -0.005813025
## concentration  0.5096999  0.2057083  1.868919615
##
## Regression coeffs for the transition probabilities:
## --------------------------------------------------
##               1 -> 2    1 -> 3     2 -> 1     2 -> 3    3 -> 1    3 -> 2
## intercept -1.289134 -3.098638 -0.6737932 -0.9159935 -2.426424 -1.161486
```

```
##
## Transition probability matrix:
## -----------------------------
##             [,1]      [,2]       [,3]
## [1,] 0.75722019 0.2086212 0.03415866
## [2,] 0.26691128 0.5235903 0.20949839
## [3,] 0.06304689 0.2233671 0.71358598
##
## Initial distribution:
## --------------------
## [1] 1.291640e-05 9.999870e-01 6.550713e-08
```

```
# fourth set of parameters (the estimated parameters of the previous model)

mu3d <- c(14, 388, 1605)
sigma3d <- c(10, 397, 749)
angleMean3d <- c(-2.97,-0.124, -0.006)
kappa3d <- c(0.51, 0.21, 1.87)
stepPar3d <- c(mu3d, sigma3d)
anglePar3d <- c(angleMean3d, kappa3d)

# fit the fourth 3-state HMM

W0612_3d <- fitHMM(W0612Reg, nbStates = 3, stepPar0 = stepPar3d, anglePar0 = anglePar3d,
                   stepDist = "gamma", angleDist = "vm")
W0612_3d
```

```
## Value of the maximum log-likelihood: -25864.43
##
## Step length parameters:
## ----------------------
##       state 1  state 2   state 3
## mean 13.87421 388.2857 1604.8032
## sd   10.06471 396.6125  749.0181
##
## Turning angle parameters:
## ------------------------
##                  state 1    state 2      state 3
## mean          -2.9698623 -0.1243197 -0.005814957
## concentration  0.5096991  0.2057115  1.868915061
##
## Regression coeffs for the transition probabilities:
## --------------------------------------------------
##              1 -> 2    1 -> 3     2 -> 1     2 -> 3    3 -> 1   3 -> 2
## intercept -1.289122 -3.098663 -0.6737882 -0.9159975 -2.426431 -1.16148
##
## Transition probability matrix:
## -----------------------------
##            [,1]      [,2]       [,3]
## [1,] 0.7572189 0.2086233 0.03415775
## [2,] 0.2669125 0.5235901 0.20949745
## [3,] 0.0630464 0.2233683 0.71358529
##
## Initial distribution:
```

```
## --------------------
## [1] 2.255205e-06 9.999977e-01 4.853313e-09
```

Model selection and assigning behavioral states to steps

We use Akaike's Information Criterion (AIC) to determine which model has the best fit to the data.

```
AIC(W0612_2a, W0612_2b, W0612_2c, W0612_3a, W0612_3b, W0612_3c, W0612_3d)
```

```
##      Model      AIC
## 1 W0612_3b 51768.86
## 2 W0612_3a 51768.86
## 3 W0612_3d 51768.86
## 4 W0612_3c 51768.86
## 5 W0612_2b 52491.27
## 6 W0612_2a 52491.27
## 7 W0612_2c 52491.27
```

All 3-state models have an equally good fit, so we arbitrarily select the final model as the "best" model.

```
# store behavioral states in new data column for best model

W0612Reg$state <- viterbi(W0612_3d)

# extract date from GMT.date into individual date column

W0612Reg$date <- as.Date(W0612Reg$GMT.date)

# get step length and turning angle means for each behavior state

bxStepMeans <- aggregate(step ~ state, W0612Reg, mean)
bxStepMeans
```

```
##   state       step
## 1     1   13.69453
## 2     2  371.89280
## 3     3 1618.44929
```

```
bxTurnMeans <- aggregate(angle ~ state, W0612Reg, mean)
bxTurnMeans
```

```
##   state       angle
## 1     1 -0.16628247
## 2     2 -0.01961102
## 3     3  0.01435323
```

Diagnostic plotting of behavioral states

We constructed a dataframe that summarizes the states per day before plotting.

```
allBx <- summaryBy(state ~ date + state + ID, FUN=length, data = W0612Reg)

prop <- function(x) x/sum(x)

allBx <- ddply(allBx, "date", transform, share = prop(state.length))

allBx$state <- as.factor(allBx$state)
```

We plot the data in ggplot to look at how the proportion of time in each state changes over time. We look only at the second and third behavioral states, as resting is not important in determining whether or not dispersal is occurring.

```
bxPlot <- ggplot(allBx, aes(x=date, y=share, group=state, colour=state)) +
  geom_hline(yintercept = 0.5, linetype = "solid", color = "grey25", size = .8) +
  geom_line(aes(linetype = state), subset(allBx, state != "1")) +
  scale_color_manual(values = c("#C4961A", "#293352")) +
  xlab("Bx State by Date") +
  ylab("Proportion of day") +
  ggtitle("Diagnostic Plot for W0612")

bxPlot
```

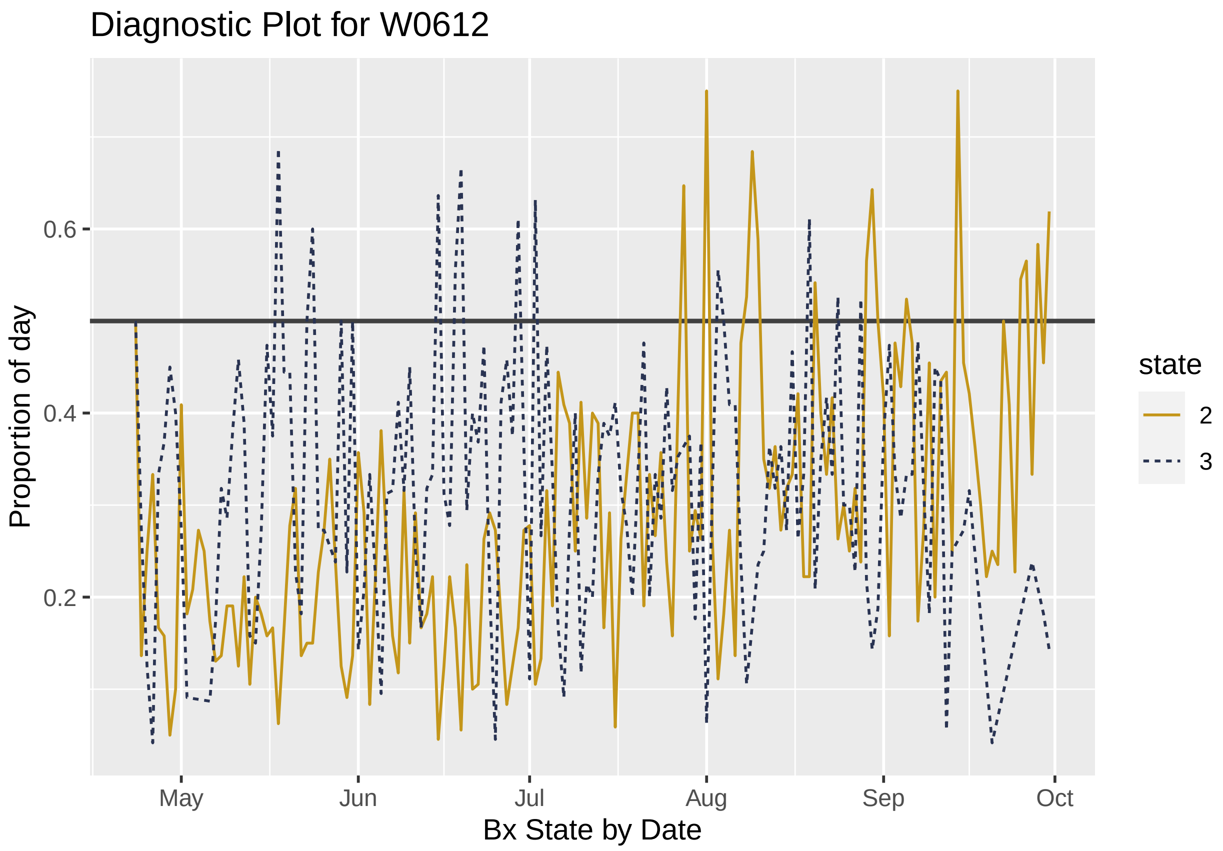


```
# interactive plot in plotly

ggplotly(bxPlot)
```

```
## Warning: `gather_()` was deprecated in tidyr 1.2.0.
## Please use `gather()` instead.
## This warning is displayed once every 8 hours.
## Call `lifecycle::last_lifecycle_warnings()` to see where this warning was generated.
```

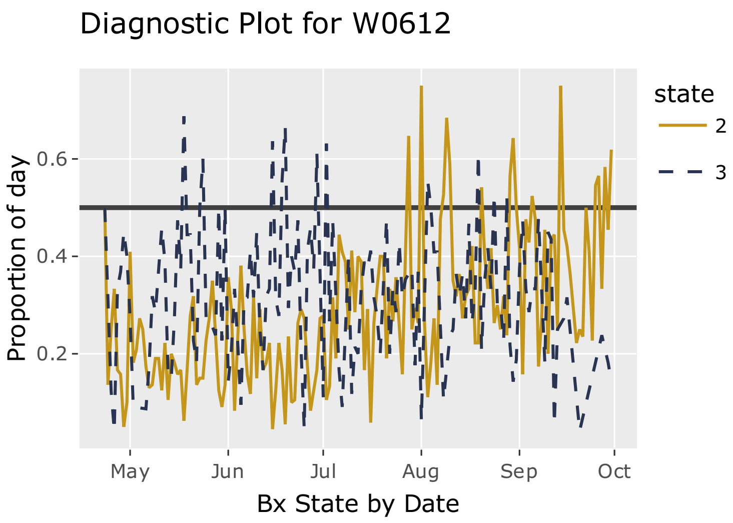

Find the dates where the individual spends at least half of the day in the third behavior, representing a dispersal state.

```r
dates <- allBx[allBx$state == "3" & allBx$share >= 0.5, ]
dates
```

```
##           date state    ID state.length     share
## 2   2009-04-23     3 W0612            1 0.5000000
## 74  2009-05-18     3 W0612           11 0.6875000
## 89  2009-05-23     3 W0612           10 0.5000000
## 92  2009-05-24     3 W0612           12 0.6000000
## 106 2009-05-29     3 W0612           12 0.5000000
## 112 2009-05-31     3 W0612           11 0.5000000
## 157 2009-06-15     3 W0612           14 0.6363636
## 166 2009-06-18     3 W0612           10 0.5555556
## 169 2009-06-19     3 W0612           12 0.6666667
## 199 2009-06-29     3 W0612           11 0.6111111
## 208 2009-07-02     3 W0612           12 0.6315789
## 302 2009-08-03     3 W0612           10 0.5555556
## 305 2009-08-04     3 W0612           11 0.5000000
## 349 2009-08-19     3 W0612           11 0.6111111
```

```
## 364 2009-08-24     3 W0612           10 0.5263158
## 376 2009-08-28     3 W0612           11 0.5238095
```

The final step was completed in the geographic information system (GIS) software, QGIS. We looked at a shapefile of the geolocations classified by behavior states. Based on the plots, date summaries, and inspection in QGIS, we selected 29 April - 02 July as the dates the individual was dispersing.

---

* geolocations have been anonymized; their spatial relationships remain unchanged

References

Zucchini, W. and MacDonald, I.L. 2009. Hidden Markov Models for Time Series: An Introduction Using R. Chapman & Hall (London).
