# Supplementary material for: Movement and habitat selection of a large carnivore in response to human infrastructure differs by life stage
Source: Mov Ecol. 2022 Nov 29;10:52. doi: 10.1186/s40462-022-00349-y (PMC9706841; doi:10.1186/s40462-022-00349-y)
Supplement: Supplementary file 2 — Additional file 2: Figure S1. The number of GPS locations at given hours of the day prior to bed removal (A) and after bed removal (B). Figure S2. Coefficient plots from resource selection functions (A) and the integrated step selection analysis (B). Yellow color indicate dispersing males and blue color indicate resident males. Table S1. The mean availability coefficient for the weighted linear models calculating the population estimates. Significant coefficients are indicated in bold and indicate a functional response, i.e. that the effect of the covariate variates with the availability. “D2.” is an abbreviation for “distance to”. [file 40462_2022_349_MOESM2_ESM.docx]

**Supplemental information**

**
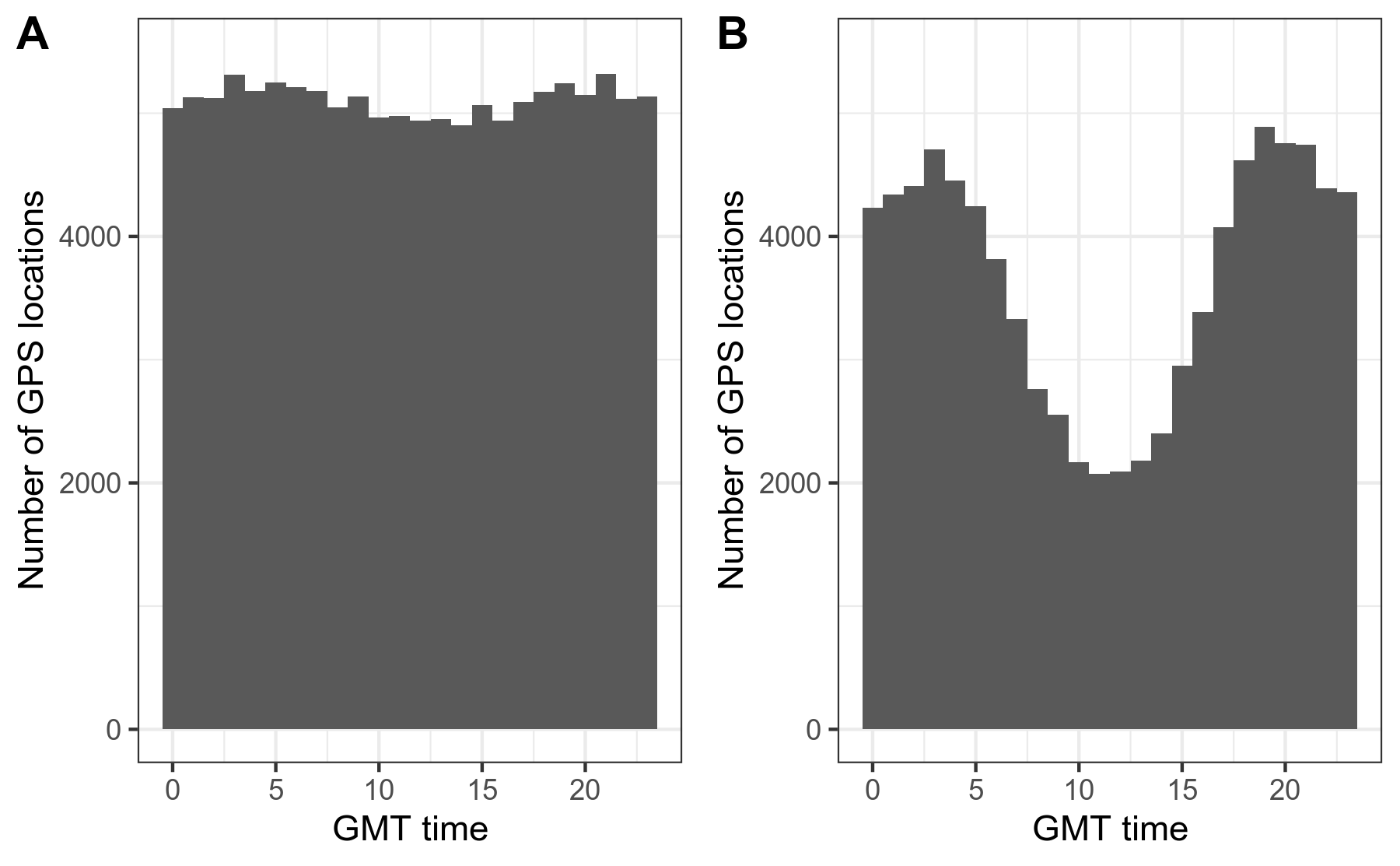
**

**Figure S1.** The number of GPS locations at given hours of the day prior to bed removal (A) and after bed removal (B).


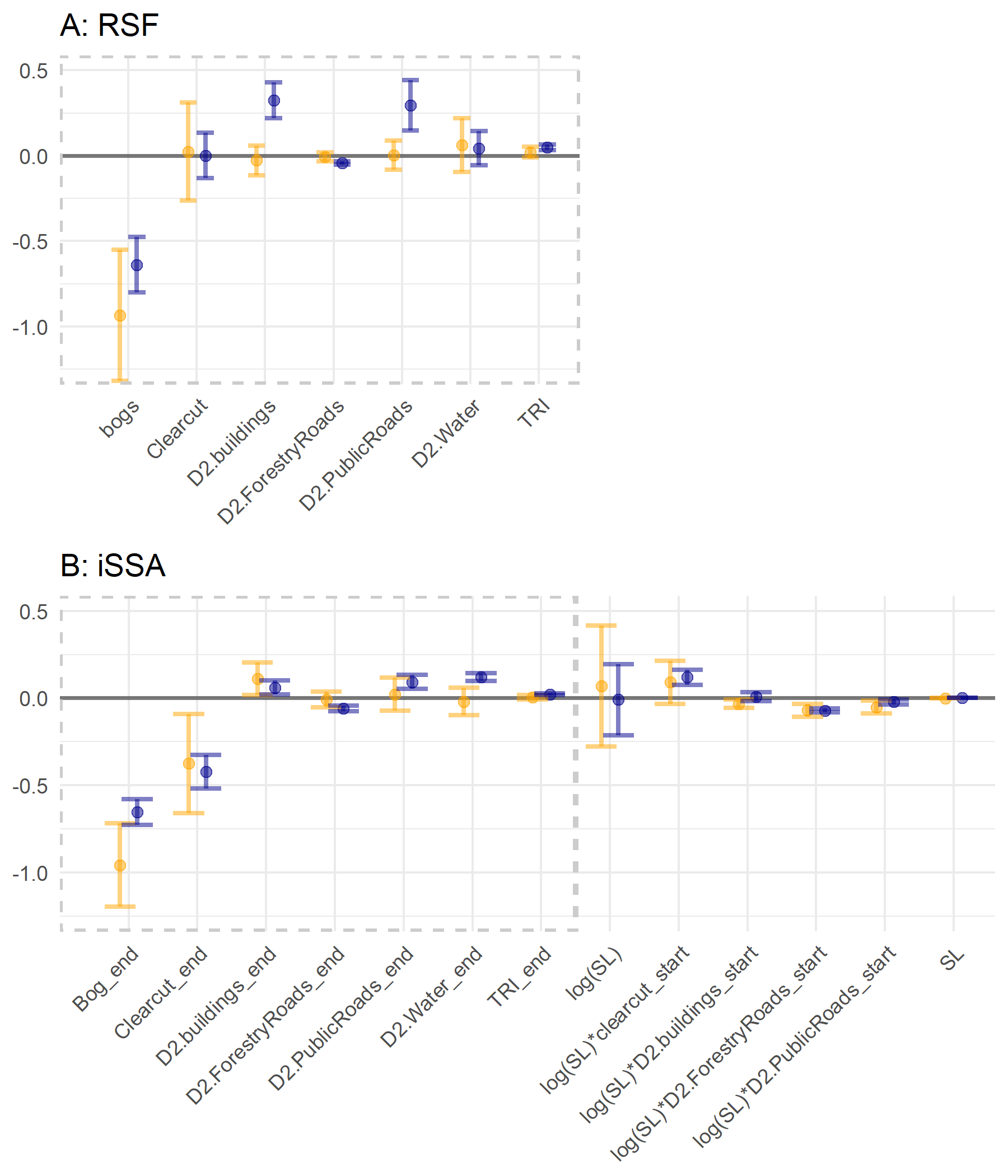


**Figure S2.** Coefficient plots from resource selection functions (A) and the integrated step selection analysis (B). Yellow color indicate dispersing males and blue color indicate resident males.

**Table S1.** The mean availability coefficient for the weighted linear models calculating the population estimates. Significant coefficients are indicated in bold and indicate a functional response, i.e. that the effect of the covariate variates with the availability.

|  |  |  | Resident | | | Dispersal | | |
| --- | --- | --- | --- | --- | --- | --- | --- | --- |
|  |  |  |  | 95% Confidence interval | |  | 95% Confidence interval | |
|  | category | coefficient (response) | mean availability (coefficient) | low | high | mean availability (coefficient) | low | high |
|  |  |  |  |  |  |  |  |  |
| *Resource Selection Function* | | |  |  |  |  |  |  |
|  | habitat selection | Bog | 31.418 | -61.067 | 123.902 | -102.111 | -238.085 | 33.863 |
|  | habitat selection | Clearcut | 1.909 | -18.175 | 21.993 | 43.935 | -18.430 | 106.301 |
|  | habitat selection | D2.buildings | -4.356 | -15.618 | 6.907 | -3.759 | -10.895 | 3.377 |
|  | habitat selection | D2.ForestryRoads | -0.102 | -0.752 | 0.547 | -0.179 | -1.936 | 1.579 |
|  | habitat selection | D2.PublicRoads | 4.708 | -9.805 | 19.221 | -3.168 | -9.237 | 2.901 |
|  | habitat selection | D2.Water | -8.026 | -25.098 | 9.047 | -3.862 | -23.778 | 16.055 |
|  | habitat selection | TRI | 0.063 | -0.546 | 0.673 | 0.065 | -0.783 | 0.913 |
|  |  |  |  |  |  |  |  |  |
| *Integrated Step Selection Analysis* | | |  |  |  |  |  |  |
|  | habitat selection | Bog_end | -0.729 | -2.932 | 1.475 | 0.214 | -5.224 | 5.652 |
|  | habitat selection | Clearcut_end | **8.221** | **4.026** | **12.416** | **19.160** | **8.428** | **29.891** |
|  | habitat selection | D2.buildings_end | 0.021 | -0.137 | 0.179 | 0.099 | -0.061 | 0.260 |
|  | habitat selection | D2.ForestryRoads_end | 0.062 | -0.016 | 0.139 | -0.002 | -0.208 | 0.205 |
|  | habitat selection | D2.PublicRoads_end | **0.168** | **0.080** | **0.256** | -0.083 | -0.215 | 0.049 |
|  | habitat selection | D2.Water_end | 0.067 | -0.018 | 0.152 | 0.298 | -0.123 | 0.719 |
|  | habitat selection | TRI_end | -0.001 | -0.005 | 0.003 | **0.013** | **0.003** | **0.022** |
|  | movement | log(SL) | -0.024 | -0.048 | 0.001 | 0.009 | -0.005 | 0.022 |
|  | movement | log(SL)*clearcut_start | -0.621 | -2.150 | 0.908 | 1.193 | -1.437 | 3.824 |
|  | movement | log(SL)*D2.buildings_start | **-0.121** | **-0.215** | **-0.027** | **-0.074** | **-0.121** | **-0.027** |
|  | movement | log(SL)*D2.ForestryRoads_start | 0.034 | -0.015 | 0.083 | -0.010 | -0.173 | 0.154 |
|  | movement | log(SL)*D2.PublicRoads_start | -0.007 | -0.034 | 0.021 | **-0.057** | **-0.110** | **-0.004** |
|  | movement | SL | 0.000 | 0.000 | 0.000 | 0.000 | 0.000 | 0.000 |
